# Tasks activating the default mode network map multiple functional systems

**DOI:** 10.1007/s00429-022-02467-0

**Published:** 2022-02-18

**Authors:** Lorenzo Mancuso, Sara Cavuoti-Cabanillas, Donato Liloia, Jordi Manuello, Giulia Buzi, Franco Cauda, Tommaso Costa

**Affiliations:** 1 FOCUS Lab Department of Psychology, University of Turin, Via Giuseppe Verdi 10, 10124 Turin, Italy; 2 Department of Physics, University of Turin, Turin, Italy; 3 GCS-fMRI, Koelliker Hospital and Department of Psychology, University of Turin, Turin, Italy

**Keywords:** Activation likelihood estimation, Task-induced deactivations, DMN, Semantics, Insula

## Abstract

Recent developments in network neuroscience suggest reconsidering what we thought we knew about the default mode network (DMN). Although this network has always been seen as unitary and associated with the resting state, a new deconstructive line of research is pointing out that the DMN could be divided into multiple subsystems supporting different functions. By now, it is well known that the DMN is not only deactivated by tasks, but also involved in affective, mnestic, and social paradigms, among others. Nonetheless, it is starting to become clear that the array of activities in which it is involved, might also be extended to more extrinsic functions. The present meta-analytic study is meant to push this boundary a bit further. The BrainMap database was searched for all experimental paradigms activating the DMN, and their activation likelihood estimation maps were then computed. An additional map of task-induced deactivations was also created. A multidimensional scaling indicated that such maps could be arranged along an anatomo-psychological gradient, which goes from midline core activations, associated with the most internal functions, to that of lateral cortices, involved in more external tasks. Further multivariate investigations suggested that such extrinsic mode is especially related to reward, semantic, and emotional functions. However, an important finding was that the various activation maps were often different from the canonical representation of the resting-state DMN, sometimes overlapping with it only in some peripheral nodes, and including external regions such as the insula. Altogether, our findings suggest that the intrinsic–extrinsic opposition may be better understood in the form of a continuous scale, rather than a dichotomy.

**Supplementary Information:**

The online version contains supplementary material available at 10.1007/s00429-022-02467-0.

## Introduction

Network neuroscience has partitioned the human connectome (Sporns et al. 2005) into a set of canonical networks (Damoiseaux et al. 2006; De Luca et al. 2006; Laird et al. 2011; Yeo et al. 2011). Among these, the Default Mode Network (DMN) is given special attention. At the time of writing, 6238 published papers were returned by the PubMed search “default mode network”, while only 2951 records for “salience network”, and 3329 for “frontoparietal network”. At least in part, the interest for the DMN likely stems from its clinical relevance, since it is found to be altered in a wide range of diseases across psycho- and neuropathology (Mohan et al. 2016; Sha et al. 2018, 2019). However, despite the attention received by the scientific community, the function of DMN remains unclear. As a matter of fact, its elusiveness might be what makes investigating such brain system so compelling.

Initially, the DMN was outlined as a network specifically related to the resting state. Its first image comes from a meta-analysis by Shulman et al. (1997) about the so-called Task-Induced Deactivations (TID), depicting the areas consistently deactivated during attention-demanding tasks. Afterwards, trying to better characterize the concept of deactivation (Raichle and Snyder 2007), Raichle et al. (2001) observed that the metabolism was mostly uniform across the brain during resting state. For this reason, they suggested that the brain at rest was in a state of physiological baseline (Raichle et al. 2001; Gusnard and Raichle 2001), a *default mode* that represents a form of tonic activation for those regions commonly deactivated by tasks (Raichle et al. 2001). Finally, Greicius et al. (2003) proved that such default mode system was indeed a network, denoted by functional connectivity (FC) at rest. Furthermore, the DMN functional signal was reported to be negatively correlated with the signal of task-positive regions at rest (Fox et al. 2005a; Uddin et al. 2009), as well as during a task and with the experimental model itself (Greicius and Menon 2004; Golland et al. 2007; Lin et al. 2011; Newton et al. 2011). However, this *rest*-*task* distinction has soon shown its limitations, in favor of an *internal*–*external* characterization of different modes of cognition (Fransson 2005; Buckner et al. 2008; Spreng 2012; Dixon et al. 2014).

From the early beginning of the investigations, it was observed that the DMN is not deactivated by any task, as self-referential and emotional paradigms activated it (Gusnard et al. 2001). Since those observations, many further functions were shown to be associated with this network. Other than self-referential and emotional processes (Fossati et al. 2003; Ochsner et al. 2004, 2005; Northoff and Bermpohl 2004; Northoff et al. 2006; Buckner and Carroll 2007; Uddin et al. 2007; D’Argembeau et al. 2010; Denny et al. 2012; Molnar-Szakacs and Uddin 2013; Engen et al. 2017; Satpute and Lindquist 2019; Fingelkurts et al. 2020; Knyazev et al. 2020), the DMN turned out to be related to memory and mental time-travel (Cabeza et al. 1997; Svoboda et al. 2006; Schacter et al. 2007, 2008; Addis et al. 2007; Foster et al. 2012; Yang et al. 2013; Rugg and Vilberg 2013; Spreng et al. 2015; Kim 2016; Murphy et al. 2018), mental simulation and scene construction (Hassabis and Maguire 2007; Hassabis et al. 2007; Spreng and Grady 2010; Gerlach et al. 2011), Theory of Mind (ToM) and social cognition (Saxe and Kanwisher 2003; Rilling et al. 2004; Ruby and Decety 2004; Saxe and Powell 2006; Mar 2011; Mars et al. 2012; Spreng and Andrews-Hanna 2015; Amft et al. 2015; Mwilambwe-Tshilobo and Spreng 2021), moral judgment (Greene et al. 2001; Harrison et al. 2008; Pujol et al. 2008; Bzdok et al. 2012), and semantic processing (Binder et al. 1999; Chiou et al. 2020; Evans et al. 2020; Lanzoni et al. 2020). However, most of these psychological functions can still be somewhat associated with the resting state, in the sense that these activities might be easily carried out during rest. In fact, the resting mind has not to be considered as idle (Raichle 2009), but it is continuously involved in activities collectively known as *mind-wandering* (Giambra 1989; Spiers and Maguire 2006; Mason et al. 2007; Fox et al. 2015; Seli et al. 2016). During mind-wandering, the brain is expected to engage in DMN-related functions such as remembering the past, imagining the future, thinking about others, and displacing the self in imaginary situations (Andreasen et al. 1995; Andrews-Hanna 2012; Andrews-Hanna et al. 2014; Christoff et al. 2016), the same functions typically associated to the DMN. When some of these activities intrude into the execution of an attentional task as *stimulus-independent thoughts*, the task performance may be affected by errors (Sonuga-Barke and Castellanos 2007; Smallwood et al. 2008; Prado and Weissman 2011; Kam and Handy 2013). Likewise, expected task-related activations may be found disrupted, and DMN areas activated (Weissman et al. 2006; Mason et al. 2007; Li et al. 2007; Eichele et al. 2008; Kam et al. 2011).

Still, these observations are consistent with a *rest*-*task* dichotomy, and do not really suggest a proactive role for the DMN. In contrast, it has been proposed that, during task, DMN-driven *stimulus-oriented thoughts* may possibly appear (Mantini and Vanduffel 2013; Sormaz et al. 2018) with the purpose of supporting task performance (Gilbert et al. 2007). As a matter of fact, the DMN has been implicated in problem-solving and creativity (Kounios et al. 2006; Gerlach et al. 2011; Abraham et al. 2012; Ellamil et al. 2012; Jung et al. 2013; Benedek et al. 2014; Mayseless et al. 2015; Marron et al. 2018; Huo et al. 2020), suggesting that its activity can also be engaged in external demands. Indeed, DMN activations can enhance cognitive control during extrinsic tasks requiring internal mentation (Spreng et al. 2014), possibly in coordination with frontoparietal control systems (Spreng et al. 2010; Cocchi et al. 2013; Gerlach et al. 2014). Furthermore, the anteromedial prefrontal cortex (amPFC), node of the DMN, was found to be activated in monitoring the external environment, contributing to faster reaction times (Gilbert et al. 2005, 2006). It also seems that the DMN is recruited in switching tasks, in the case of a demanding shift from a cognitive context to a different one (Crittenden et al. 2015). Moreover, the DMN was also found to be activated both in decision-making (Smith et al. 2021) and when subjects have to automatically apply learned rules (Vatansever et al. 2017).

The critical role of DMN for task execution is highlighted not only by activation studies, but also by functional connectivity investigations that questioned the supposed DMN anticorrelation with task execution and task-positive regions. It has been shown that nodes of the DMN are positively correlated with task-positive areas during acquisition and retrieval phases of a working memory task (Piccoli et al. 2015), as well as during the preparation phase (Koshino et al. 2011, 2014). In addition, during such tasks, the connectivity within the DMN was found to be correlated with behavioral performance (Hampson et al. 2006). Moreover, the sign and the strength of the correlations between DMN and task-positive regions remarkably varies between the nodes of these networks, and according to the tasks and the different resting-state time epochs (Chang and Glover 2010; Bluhm et al. 2011; Leech et al. 2011; Elton and Gao 2015; Dixon et al. 2017; Denkova et al. 2019). At the subject level, interactions between DMN and attentional and control networks were found during a semantic memory retrieval task (Fornito et al. 2012). In sum, there is considerable evidence that the DMN functionality is crucial, not only for internal mind-wandering, but also for the execution of extrinsic activities.

Just as its role in human cognition, the anatomical and topological representation of the DMN has proven to be puzzling. From a theoretical point of view, the DMN could be described as a whole and unfractionated network, with hub nodes in the posterior cingulate cortex (PCC) and medial prefrontal cortex (mPFC) and more peripheral nodes in the medial and lateral temporal lobe, angular gyrus (AG), dorsolateral prefrontal cortex (dlPFC), and inferior frontal gyrus (IFG) (Buckner et al. 2008; Yeo et al. 2011). Nonetheless, it is widely acknowledged that it can be further divided into subnetworks (Abou-Elseoud et al. 2010; Abou Elseoud et al. 2011; Yeo et al. 2011; Shirer et al. 2012; Ray et al. 2013). In this regard, Andrews-Hanna et al. (2010) reported that the subdivisions of such network might have different functions. In particular, they identified two subsystems structured around a midline core: the former, formed by the PCC was thought to be involved in self-referential processes, and the latter, composed by mPFC, has considered to be related to future-oriented thoughts (Andrews-Hanna et al. 2010). What is more, the midline core itself might be a fractionated structure. In fact, by analyzing single-subject data with minimal spatial smoothing, Braga and colleagues (Braga and Buckner 2017; Braga et al. 2019; DiNicola et al. 2020) have recently found that the DMN seems to be composed of two parallel and interdigitated networks, interleaved even within PCC and mPFC. The two subsystems were found to be related to different roles: one to social cognition and the other one to mnestic functions (DiNicola et al. 2020). Similarly, Wang et al. (2020) parcellated the DMN nodes into different parts, each one associated with a specific functional profile. Likewise, Gordon et al. (2020) were able to divide the individuals’ DMN into nine subnetworks showing differential task engagement. Thus, the most recent developments in the research of the DMN are indicating that such network, far from being a monolithic entity, consists of multiple systems with intersecting functions and anatomies (Buckner and DiNicola 2019).

The present study aims to delve into this matter, using a coordinate-based meta-analytical methodology to investigate the functions related to the activity of the DMN regions and the spatial variability associated with them. The use of a meta-analytical approach allows to overcome the heterogeneity of results, a typical issue of neuroimaging experiments (Botvinik-Nezer et al. 2020). After all, the DMN research has a long meta-analytical tradition. In fact, the first images of the network come from meta-analyses of TID (Shulman et al. 1997; Mazoyer et al. 2001), subsequently confirmed by an Activation Likelihood Estimation (ALE) meta-analysis by Laird et al. (2009). Another ALE study (Schilbach et al. 2012) showed that the areas of TID, social cognition, and emotional processing converged on PCC and mPFC. Similarly, an ALE meta-analysis from Spreng et al. (2009) noted a correspondence between autobiographical memory, spatial navigation, theory of mind activations, and TID. It could be said that these two latter studies used a meta-analytic approach to put forward a consistent and comprehensive view of DMN functions. On the contrary, the current study wants to highlight the functional variety of the DMN.

To make these assessments, we performed a Paradigm Analysis (Lancaster et al. 2012), capitalizing on the BrainMap database and on its taxonomy of behavioral ontologies (Fox and Lancaster 2002; Fox et al. 2005b; Laird et al. 2005). This allowed us to identify the task categories significantly associated with the network in a data-driven fashion. Activation coordinates of such paradigms were then obtained from the same database, and used to perform an ALE meta-analysis for each one of them. As indicated by Raichle et al. (2001), TID correspond to rest tonic activations, and it has been suggested that the rest should be seen just as another active state (Buckner et al. 2013). Thus, a TID ALE map was calculated to represent the DMN configuration during resting state. This also constitutes a replication of Laird et al. (2009) with a larger database and updated algorithms. The resulting set of maps underwent a series of analyses, namely, Multidimensional Scaling (MDS), Principal Component Analysis (PCA), and Independent Component Analysis (ICA). These were to explain the DMN task-based variability in the form of axes along which the network arranges itself to meet external demands. We expected that different operative domains related to the DMN would recruit distinctive sets of areas, including some regions typically assigned to other resting-state networks (RSNs). Given the wide range of functions implicated with the mPFC (Delgado et al. 2016; Schneider and Koenigs 2017; Hiser and Koenigs 2018; Lieberman et al. 2019; Toro-Serey et al. 2020), we anticipated that this region would express a large variability in its activations, possibly organized in a rostral-caudal arc revolving around the callosal genu (Amodio and Frith 2006). The PCC might show some internal differentiation as well (Leech et al. 2011).

## Methods

### Paradigm Analysis

The Paradigm Analysis (Lancaster et al. 2012), as implemented in the dedicated plugin for Mango (http://ric.uthscsa.edu/mango/) (Lancaster et al. 2010, 2011), was performed to get the profile of involvement of the DMN with different fMRI paradigms. The BrainMap database is coded with an articulated cognitive, anatomic and operational taxonomy (Fox et al. 2005b). In particular, each experimental contrast is characterized by the domain of the task utilized. A complete list of paradigms with detailed definitions can be found at: http://www.brainmap.org/taxonomy/paradigms.html. Given a region of interest (ROI), the Paradigm Analysis leverages on the BrainMap database to test if executing a task significantly activates that area, versus a null model of spatial uniformity of that paradigm’s activations. The output of the technique is a series of z-scores for each paradigm, whose significance is set above the Bonferroni-corrected threshold of *z* = 3.3. A paradigm being significant in a ROI does not mean that it activates only that area and not the rest of the brain, but that there is a density of activation higher than chance within the mask. Thus, we are not looking for the tasks activating specifically the DMN, but simply the task activating the DMN more than chance, while other RSNs might be activated as well.

Of note, numerous and different functional parcellations of the human brain exist, and there are no methodological criteria or gold standard to prefer one to the others (Eickhoff et al. 2015; Arslan et al. 2018). Therefore, to maximize the representativity of our results, three different masks of the DMN obtained with different methodologies were fed to the Paradigm Analysis. The paradigms that were found significantly associated with at least two out of these three masks, were taken in consideration in the subsequent analyses. The first ROI was extracted from the seven Network parcellation by Yeo et al. (2011), which was produced with a clustering algorithm. The second one was derived from the ICA by Shirer et al. (2012), merging the originally split ventral and dorsal components of the DMN. Lastly, we selected the DMN from the CAREN 5 atlas by Doucet et al. (2019), which was obtained by the consensus between six different parcellations. Since the Paradigm Analysis tool works in Talairach space, the three masks were registered to Talairach using FLIRT (Jenkinson et al. 2002).

### Activation likelihood estimation and fail-safe analysis

To trace the studies related to the 8 significant paradigms identified by such consensus approach (see Sect. 3.1), the software Sleuth has then been used to search the BrainMap functional database (Fox and Lancaster 2002; Fox et al. 2005b; Laird et al. 2005). For each one of the paradigms individuated with the method detailed above, the queries were composed as follows:

*[Experiment Context is Normal Mapping] AND [Experiment Activation is Activations Only] AND [Experiment Paradigm Class is …]*

where the latter field was completed with the given paradigm. Furthermore, to obtain the TID, we replicated the search used by Laird et al. (Laird et al. 2009):

*[Experiment Context is Normal Mapping] AND [Experiment Activation is Deactivations Only] AND [Experiment Control is Low Level]*

Coordinates were exported by Sleuth in Talairach space. To minimize within group effects and ensure independence between the observations (Turkeltaub et al. 2012; Müller et al. 2018), the experimental contrasts calculated on the same group of subjects were merged in a single set of foci, using the pertaining option in the tool.

Each one of the resulting lists of coordinates was then fed to GingerALE 3.0.2 (Turkeltaub et al. 2002; Eickhoff et al. 2009) to calculate its ALE map. A family wise error (FWE) correction for multiple comparisons was adopted (Eickhoff et al. 2012), with cluster-level inference of *p* < 0.05 and a cluster-forming threshold of *p* < 0.001.

To take into account the file-drawer effect, a Fail-Safe procedure was implemented (Acar et al. 2018). The file-drawer problem, also known as publication bias, hypothesizes the existence of unpublished null results that would reduce the representativity of a meta-analysis (Rosenthal 1979). The Fail-Safe algorithm (https://github.com/NeuroStat/GenerateNull) proposed by Acar et al. (2018) generates a given number of experiments having random foci, to be added to the dataset to be tested. The purpose of these random experiments, which do not corroborate our findings, is to simulate unpublished results. The number of foci and subjects of those experiments were such to match the distribution they had in the original data. Samartsidis et al. (2020) estimated that the file-drawer effect of the BrainMap database amounts to 6%. Thus, we decided to perform a series of Fail-Safe analyses adding the 6% and the 60% of random experiments to each dataset, so as to evaluate the robustness of our results. The outputs were two series of maps showing the activations that were still significant after the injection of the specified level of noise.

### Data reduction techniques

The subsequent analyses were carried out in Python, using the NiBabel 3.2.1 package (Brett et al. 2020) to access the NIfTI file format, the NumPy library (Harris et al. 2020) to calculate the Pearson correlation coefficients and scikit-learn 0.24.1 (Pedregosa et al. 2011) to compute MDS, PCA and ICA. The Bartlett’s test and the Kaiser–Meyer–Olkin index were calculated with FactorAnalyzer (https://factor-analyzer.readthedocs.io/en/latest/index.html). Matplotlib (Hunter 2007) was used for visualizations.

To start with, the 9 ALE maps (8 paradigms and the TID) were vectorized using a Talairach standard as brain mask with 2 mm^3^ voxel size (https://www.brainmap.org/ale/colin_tlrc_2x2x2.nii.gz), to obtain a *voxels* × *maps* matrix. To perform the MDS, a Pearson’s correlation matrix was derived first. Then, the distance measure *d*_*ij*_ between the element *i* and *j* of the dissimilarity matrix was calculated as *d*_*ij*_ = 1—*r*_*ij*_ where *r* is their Pearson’s correlation, as in Kriegeskorte et al. (2008). Such distance matrix was then fed to the scikit-learn MDS algorithm. The MDS attempts to reproduce the distances of the input matrix in an Euclidean space of reduced dimensionality that preserves the original dissimilarities as closely as possible. To avoid random rotations of the solutions, the iterative MDS algorithms were initialized using PCA scores as starting condition (Bécavin et al. 2011). We calculated the solutions with 2 and 3 dimensions, calculating the standardized stress *S* (Kruskal 1964) to evaluate the goodness-of-fit of the two solutions. The stress was computed implementing the code proposed at: https://github.com/scikit-learn/scikit-learn/pull/10168.

The *voxels* × 9 *maps* matrix was again used as input for PCA to obtain both the principal component (PC) loadings (map coefficients for each component) and scores (voxel projections on the component). The scores were then plotted on the Talairach standard to obtain PC maps. Finally, the same *voxels* × *maps* matrix was fed to the scikit-learn FastICA algorithm (Hyvärinen and Oja 1997; Hyvarinen 1999). In doing so, we obtained the voxel scores on each independent component (IC) to be plotted in Talairach space to obtain the corresponding voxel-wise maps, as well as each map’s coefficient, or loading, on each component.

### Comparison with the resting-state principal gradients

To further characterize our results, we compared our IC maps with those of the first and third principal gradients (PG) by Margulies et al. (2016). Since our maps were not restricted to the cortex, we chose to work with PG whole-brain volumes, downloaded from https://neurovault.org/collections/1598/ and converted to Talairach space with FLIRT. PG1 is known to be related to cortical hierarchy, and PG3 is taken here as another dimension of associativity. To confirm this, we performed a meta-analysis similar to that used by Margulies et al. (see Supplementary Materials for more details).

A first comparison was made correlating the ICs with the PGs, using the same mask as above (see Data reduction techniques) to exclude non-brain voxels from the computation. In addition, for a given IC and PG, we extracted the PG values corresponding to the positive part of the IC, and compared them to the remaining PG values with a two-sample *t* test (*H*_*0*_: *PG*_*IC*_ ≤ *PG*_*¬IC*_). Lastly, we portrayed the heteromodality of our ICs as the percentage of their positive part overlapping with the positive voxels of the given PG. The same analyses were performed for the ALE and PC maps as well.

## Results

### Paradigm Analyses and activation likelihood estimations

The Paradigm Analysis results obtained for the three selected DMN masks (Yeo et al. 2011; Shirer et al. 2012; Doucet et al. 2019) are presented in Table 1. These tasks were found to activate the respective DMN ROIs significantly more than by chance, that is, the activations for a given task were found within the DMN more frequently than those expected if they were distributed randomly in the brain. The three DMN masks are shown in Supplementary Fig. S1. Eight paradigms were found to be significant for at least two out of the 3 DMN masks: ToM, Semantic Monitor/Discrimination, Episodic Recall, Emotion Induction, Self-Reflection, Deception, Imagined Object/Scenes, and Reward. Most of these paradigms are related to social, mnestic, or other internal mentation functions typically associated with the DMN (Buckner et al. 2008). To the best of our knowledge, deception tasks were not previously related to the network, although the social nature of such paradigms likely justifies this result. Similarly, we are not aware of many explicit links between reward functions and DMN in the literature (Lopez-Persem et al. 2020; Martins et al. 2021), albeit there is strong evidence to associate the mPFC to such mechanisms (Xue et al. 2009; Schneider and Koenigs 2017; Hiser and Koenigs 2018; Lieberman et al. 2019). Interestingly, reasoning and problem-solving paradigms had a significant effect on the CAREN mask (Doucet et al. 2019), suggesting that the DMN might then play a role outside of what is considered internal mentation in the strictest sense.

**Table 1:** BrainMap paradigms found to be significantly associated with the DMN masks obtained by the 7 networks atlas by Yeo et al. (2011), the Independent Component Analysis by Shirer et al. (2012), and the CAREN atlas by Doucet et al. (2019). The paradigms excluded from further analysis are written in italics

Yeo et al		Shirer et al		Doucet et al	
BrainMap paradigm	*z* score	BrainMap paradigm	z score	BrainMap paradigm	*z* score
Theory of Mind	13.696	Theory of Mind	7.986	Theory of Mind	8.359
Semantic Monitor/Discrimination	5.255	Episodic Recall	5.266	Semantic Monitor/Discrimination	6.68
Episodic Recall	5.227	Self-Reflection	4.395	Episodic Recall	5.414
Emotion Induction	4.768	Emotion Induction	4.262	*Cued Explicit Recognition/Recall*	4.369
Self-Reflection	3.872	*Acupuncture*	4.043	Emotion Induction	4.303
Deception	3.69	Imagined Objects/Scenes	3.954	*Reasoning/Problem Solving*	4.112
*Passive Listening*	3.327	Reward	3.458	Reward	3.647
				Deception	3.614
				*Reading (Covert)*	3.601
				Imagined Objects/Scenes	3.537
				*Face Monitor/Discrimination*	3.536

Details about the results from Sleuth searches for the eight queries associated with each significant paradigm are presented in Table 2 and in the Prisma Flow chart in Supplementary Fig. S2. We point out that we found a limited number of experiments for the Self-Reflection condition. According to Eickhoff and colleagues (Eickhoff et al. 2016; Müller et al. 2018), at least 17 experiments should be gathered to perform a statistically sound ALE. Although the query returned 28 experiments, 7 sets of foci remained after merging them according to groups of subjects (Müller et al. 2018). To rule out possible bias induced by the inclusion of this underpowered domain, the subsequent analyses were repeated with and without the related ALE map. Since excluding this condition did not significantly influence their outcomes, we decided to keep it in the dataset.

**Table 2:** Details about the result of Sleuth queries for the main analysis

*N* experiments	*N* groups	*N* foci	*N* subjects
Theory of Mind			
218	63	1663	1127
Semantic/Monitor Discrimination			
646	205	4954	3020
Episodic Recall			
123	39	1009	566
Emotion Induction			
537	166	3575	3234
Self-Reflection			
28	7	144	140
Deception			
115	39	885	954
Imagined Object/Scenes			
120	46	1097	660
Reward			
757	199	5860	3681
Task-induced deactivations			
189	106	1665	1494

**Fig. 1:**
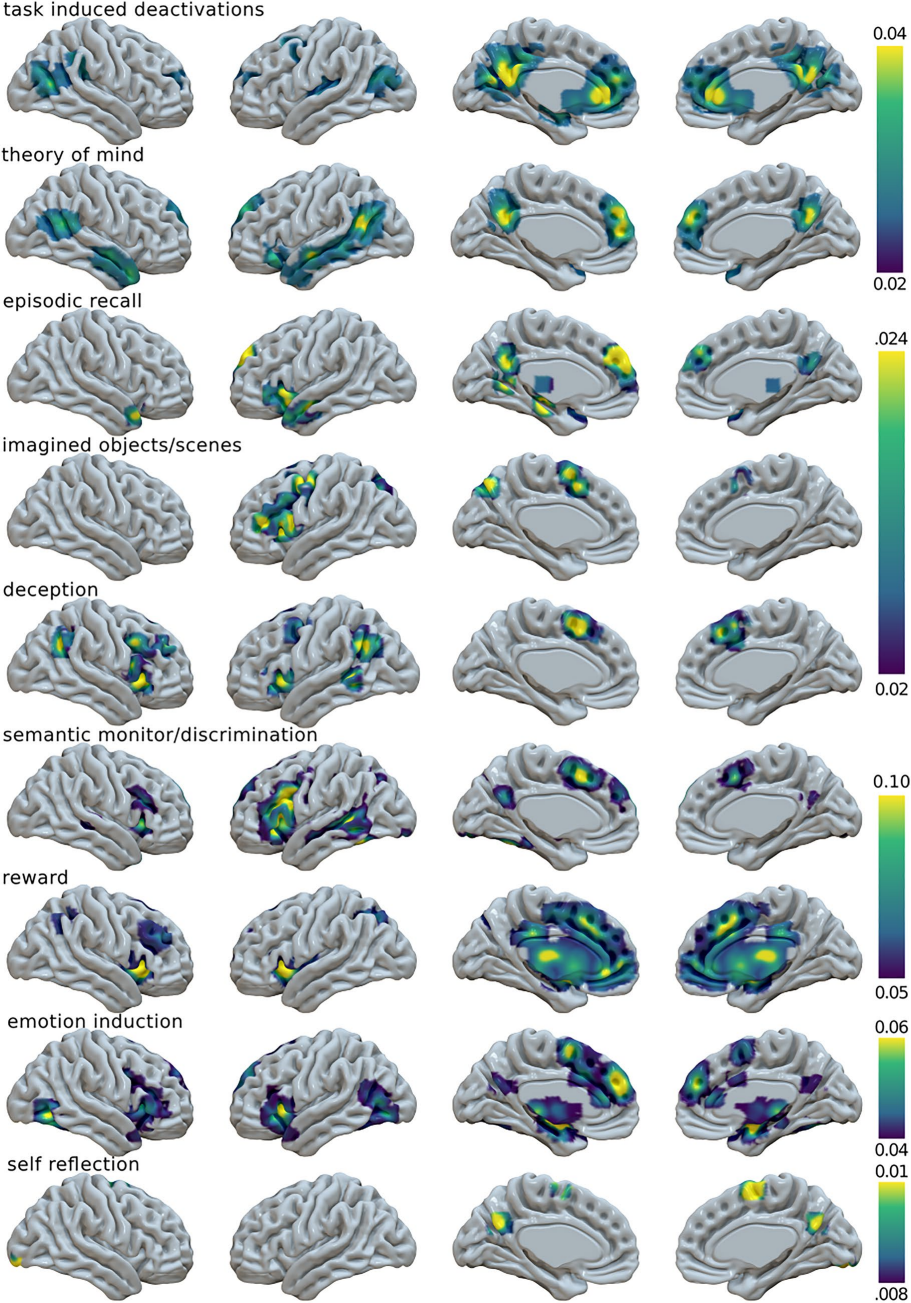
Surface mapping of the nine activation likelihood estimation maps

We also computed a TID meta-analysis, representing rest. The ALE results are presented in Fig. 1. The TID ALE map replicates the one by Laird et al. (2009), with the exception of the mPFC cluster, that we found in a more dorsal position.

As for the other maps, Episodic Recall activates the left insula along with DMN areas. Imagining objects and scenes activate the left hemisphere in the precuneus, lateral prefrontal cortex (lPFC) including IFG, and supplementary motor area (SMA). Deception tasks activate the bilateral insula, other than AG and SMA, in accordance to Farah et al. (2014). Semantic monitoring and discrimination activate the left lPFC including IFG, but also the SMA, the left lateral and medial temporal lobe, and the PCC. This map is very similar to a recent ALE about semantic control (Jackson 2021), with the exception of PCC, which is a novel result. Reward activates the basal ganglia (BG), the thalamus, the whole mPFC, and the bilateral insula. ToM activates the temporal and temporoparietal cortices along with PCC and dorsomedial PFC (dmPFC), especially on the left. Inducing emotion activates the left insula and the bilateral occipito-temporal cortices, along with typical DMN nodes. The Self-Reflection map involves PCC, the right SMA, and an occipital cluster in the fusiform gyrus. The maps obtained by the Fail-safe analyses, presented in Supplementary Fig. S3 and S4, show that our ALE maps remain substantially unchanged when accounting for the file-drawer effect.

**Fig. 2:**
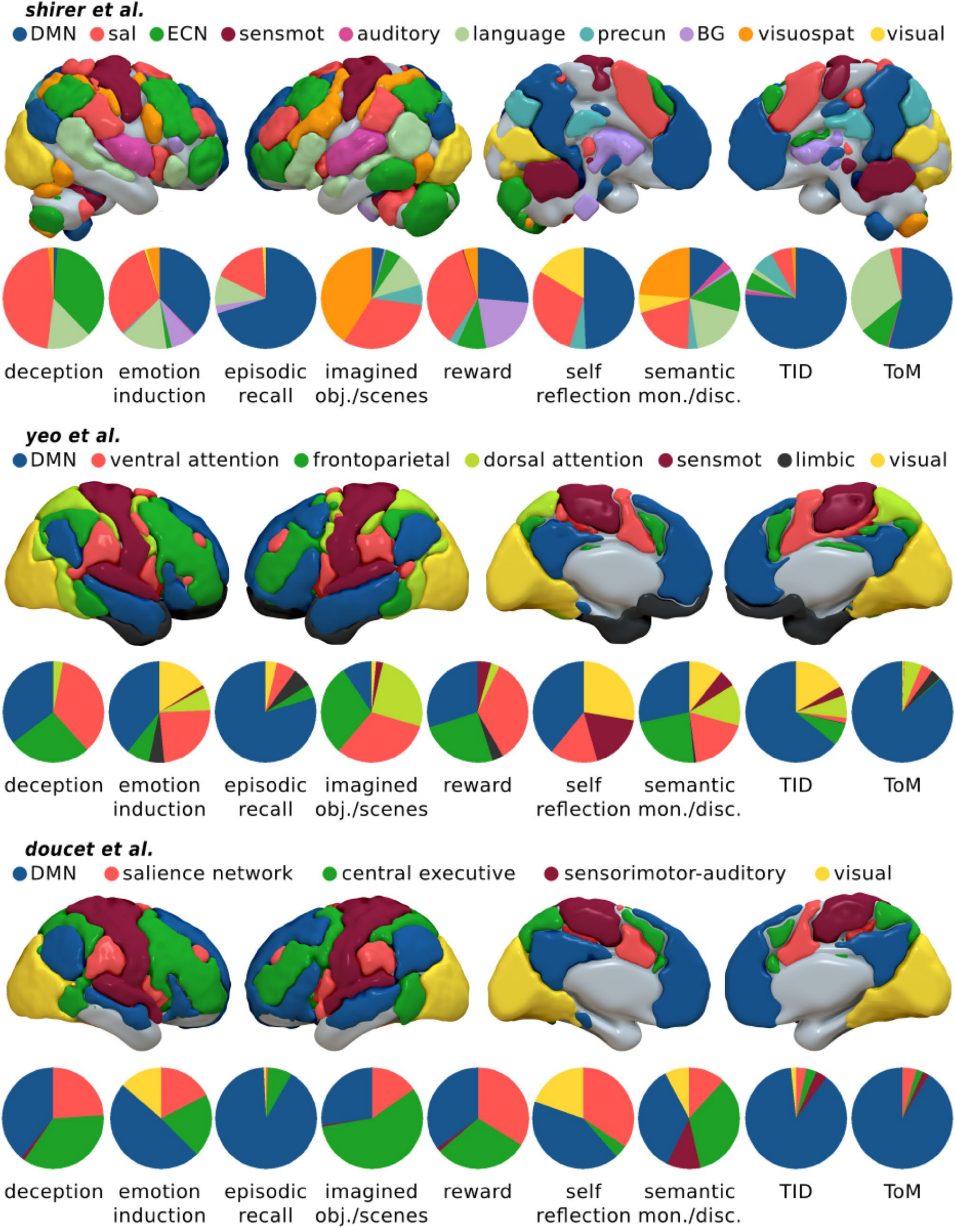
Pie charts with the proportions of each ALE voxels for each one of the resting-state networks proposed by Shirer et al., Yeo et al., and Doucet et al. As reference, each parcellation is presented in form of volume renderings. *ECN* executive control network, *sal* salience network, *precun* precuneus, *BG* basal ganglia, *visuospat* visuospatial network, *sensmot* sensorimotor network. The ECN by Shirer et al. roughly corresponds to the frontoparietal network by Yeo et al. and Doucet et al. The ventral attention network by Yeo et al. includes the salience network. The language network by Shirer et al. includes large parts of the DMN as depicted by others

In summary, few maps matched the prototypical representation of the DMN. Some of them showed either weak activation or no activation at all in the midline core, and a strong expression of lateral areas of the network such as AG, IFG, and middle temporal gyrus. In addition, the insula and SMA/dorsal ACC, hubs of the salience network (SN), were often present. Figure 2 shows the proportions of voxels of each ALE map falling within each one of the RSNs of the three functional parcellations. The overlap between the ALE maps of the main analysis and the three DMN masks is shown in Supplementary Fig. S5. The Jaccard indices between the main analysis ALE maps and the various RSN reported by Shirer et al. Yeo et al. and Doucet et al. are presented in Supplementary Table S1 and Fig. S6.

### Multidimensional scaling

**Fig. 3:**
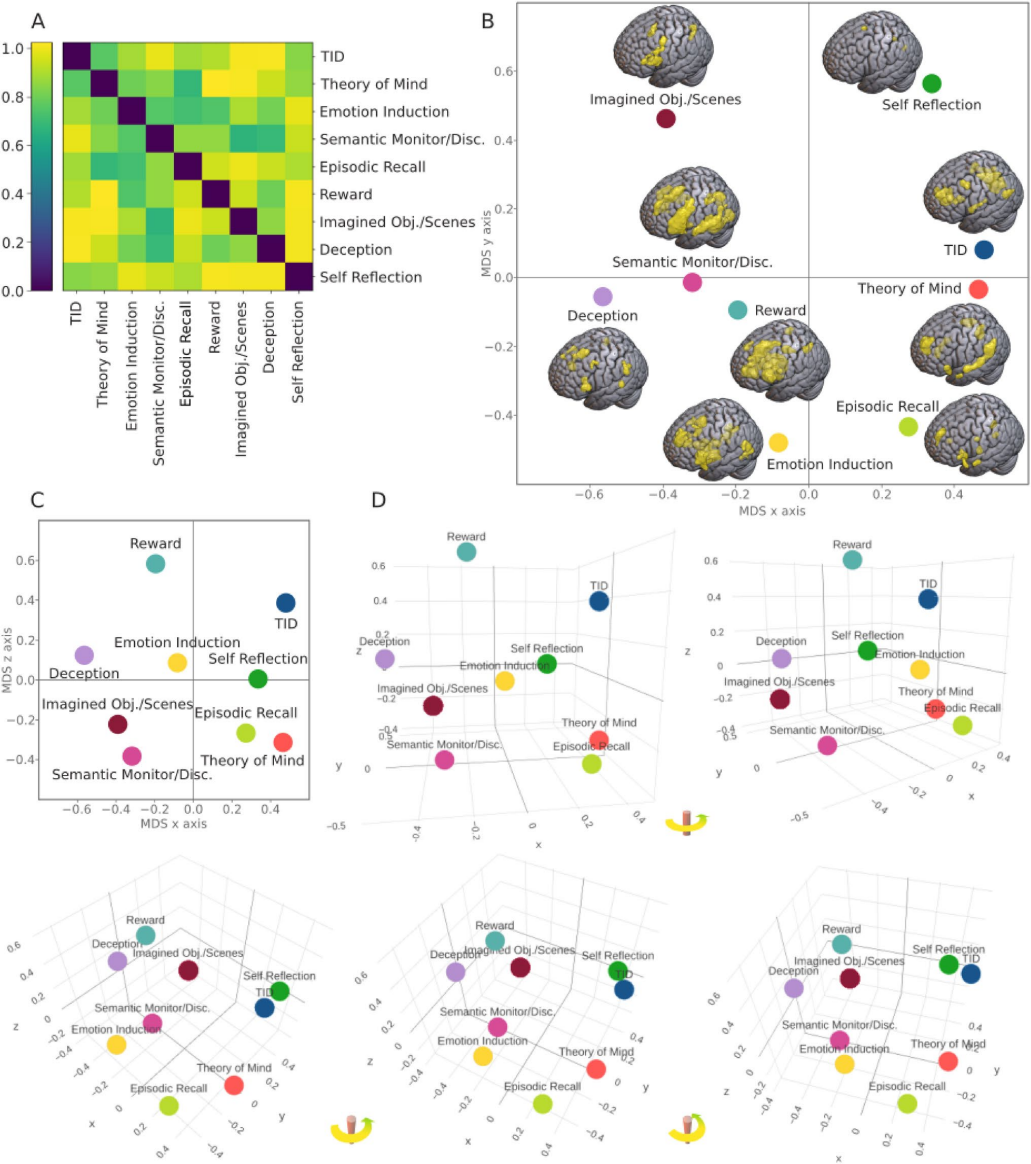
Multidimensional scaling (MDS) of the nine activation likelihood estimation (ALE) maps. A: 1 − r distance matrix of the nine ALE maps. B First two axis of the MDS 3-dimensional solution. ALEs volume mappings are shown next to their respective MDS coordinates. C First and third axis of the MDS 3-dimensional solution. D MDS 3-dimensional solution, seen from different perspectives. From left to right, the views are progressively rotated for a better understanding of tridimensionality

The standardized stress of the 2D solution is *S* = 0.25, which is considered as a “poor” solution (Kruskal 1964), while the 3D solution has a “fair” stress of *S* = 0.15. Thus, only the 3D solution will be considered between the main results. The 1 − *r* dissimilarity matrix (Kriegeskorte et al. 2008) between maps and the resulting 3-dimensional MDS can be seen in Fig. 3 (the 2D solution is presented in Supplementary Fig. S7). The MDS graphs obtained excluding the Self-Reflection condition are presented in Supplementary Fig. S8. Both with and without the Self-Reflection map, the 3D MDS solution seems to suggest a first axis standing for medial–lateral, or a core-lPFC, spatial representation. The maps found on one side of the axis (e.g., TID, ToM, and Episodic Recall) show activations in the midline DMN core, while those found on the other side (e.g., Deception, Imagined Objects/Scenes, Semantic Monitor/Discrimination) display a weaker involvement of such regions, especially PCC. Conversely, the latter have significant clusters in the lPFC and insula, and the midline activations are especially located in the SMA. The maps activating the midline core are not only those presenting more similarity with its stereotypical image, but also those whose functions were more commonly associated with the DMN (Buckner et al. 2008). As for the opposite side, these maps are related to tasks more rarely associated with the network, and they bear a similarity with the spatial distribution of SN, rather than the one of DMN. Moreover, they are reminiscent of the semantic regions (i.e., SemN) found by Chiou et al. (2020) as belonging to a more outward-leaning DMN subsystem (see also Evans et al. 2020). Hence, this anatomical midline-lateral axis could also be seen as a psychological internal/external dimension. As for the second axis, the distribution of the maps may suggest a dorsal–ventral labeling. On one side, activations are more focused in SMA, PCC, or dorsal frontal and parietal cortices. On the other side, there are insular, temporopolar, and medial temporal areas. The third axis added in the 3D solution does not have an obvious explanation. A quantitative analysis confirming our interpretation is presented in the Supplementary Materials at Table S2 and Fig. S9.

### Component decompositions

Given the lack of common ground between different DMN expressions, we performed a PCA to summarize the inter-paradigm similarities. Even if the high significance of the Bartlett’s test (*p* < 0.001) seems to suggest otherwise, the Kaiser–Meyer–Olkin index (*KMO* = 0.61) indicates a sufficient but mediocre relation between the maps. The first four principal components (PC) explain 96.3% of the variance, and loads particularly on Reward (PC1, 51.8% of explained variance), Semantic Monitor/Discrimination (PC2, 30.5%), Emotion Induction (PC3, 10.1%), TID and ToM (PC4, 4%). Supplementary Fig. S10 shows the PC maps and respective loadings. The PCA results computed excluding the Self-Reflection map were identical (not shown).

**Fig. 4:**
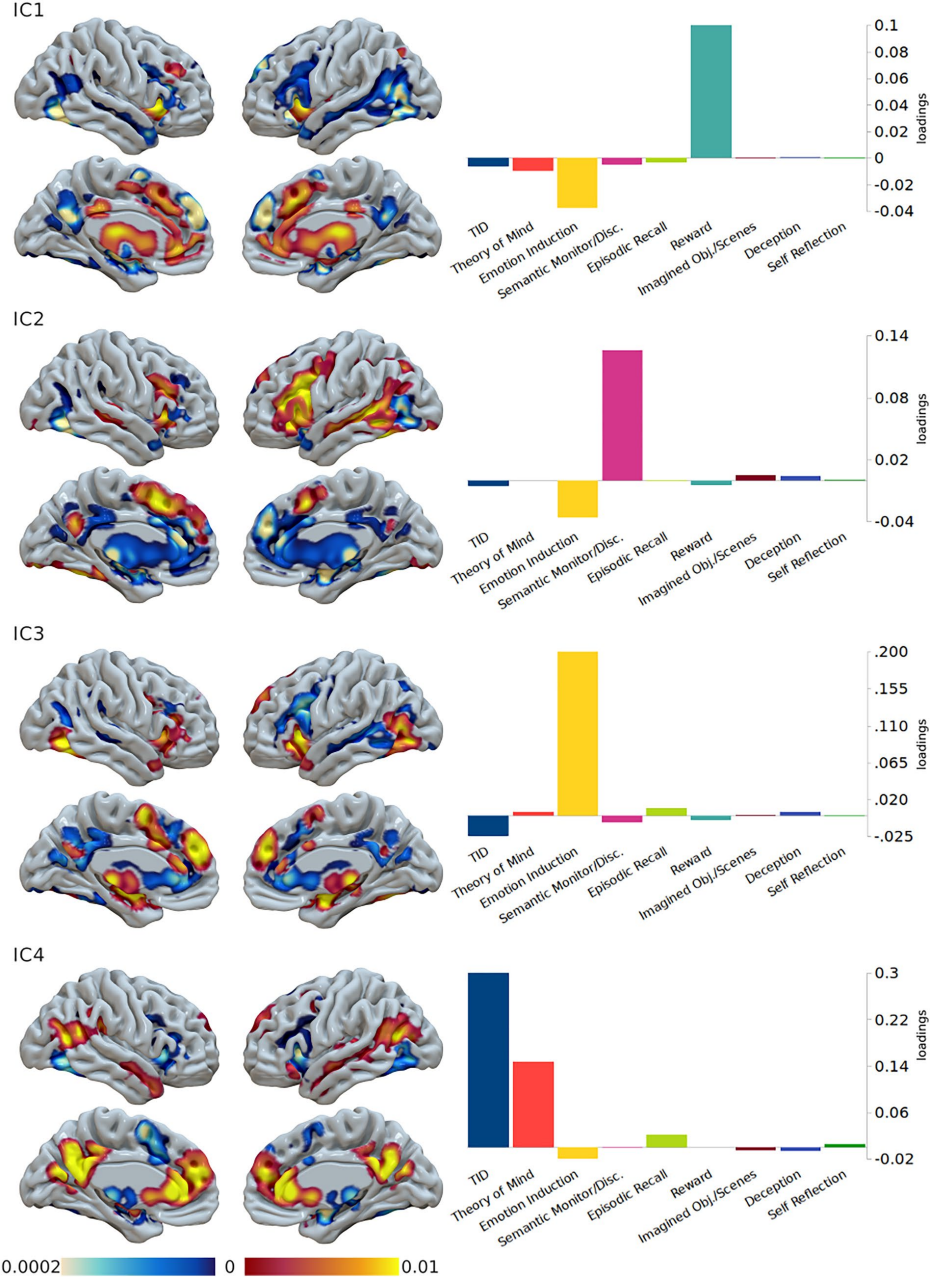
Results of the four-component solution of the Independent Component Analysis. Left: surface mapping of the voxel-wise scores. Right: weights of the unmixing matrix of each component (loadings) on each paradigm map

As the PCA indicated that four principal components provide a significant decomposition of the variance, we selected a four-component solution for the ICA as well (Fig. 4). When excluding the Self-Reflection map from the data, the ICA results did not change (not shown). The independent components (IC) were similar to those of the PCA. Thus, for an easier argumentation, we ordered them so as to match the principal ones.

IC1 is associated with Reward and shows positive coefficients in most mPFC, anterior PCC, insula, and thalamus/BG. However, it presents negative values with dmPFC, PCC proper, occipito-temporal cortices, and amygdala. IC2, related to Semantic Monitor/Discrimination, shows positive coefficients especially in the left hemisphere, in a cluster formed by the PFC (including IFG), AI, and most of the premotor cortex, as well as in the temporal cortex, SMA and PCC. Large parts of the midline display negative values, in particular the dmPFC, as the component loads negatively to Emotion Induction and this area is activated by such task (Fig. 1). IC3, associated with Emotion Induction, is positively associated with AI, temporo-occipital cortices, dmPFC, a small part of PCC, thalamus and amygdala. Negative coefficients are closely juxtaposed to positive areas, in BG, around PCC, in prefrontal and temporal cortices. IC4 is linked to TID and ToM, and it is clearly expressed in the canonical DMN regions and anticorrelated with the insula, SMA, and amygdala.

Intriguingly, when the ICA maps were fed back into a Paradigm Analysis (positive voxels only), the output returned a much longer list of significant experimental tasks (Supplementary Table S3 and Fig. S11), comprising the paradigms heavily loaded by the component. IC4 represents an exception, as it gives only few paradigms other than those found by the three original DMN masks. This is not surprising, as IC4 is associated with TID. This analysis suggests that IC1, IC2, and IC3, compared to the canonical DMN, are more closely associated with different modes of extrinsic cognition.

Exploratory analyses pointed out that solutions with more than five components produced patchy maps that may be considered as noise, or as evidence that our data have no information to be further decomposed. The five-component solution was rather similar to the one presented here, except for TID and ToM split into two different components. The ToM component involves a smaller and more dorsal mPFC cluster, and a more posterior temporoparietal cluster than the TID one (Supplementary Fig. S12). On the contrary, solutions two and three are not particularly meaningful. They showed maps similar to the canonical DMN or its anti-network, but their components were unable to load on more than a paradigm each one (Supplementary Fig. S13).

### Comparison with the resting-state principal gradients

The first PG by Margulies et al. (2016) is a non-linear component resembling the DMN and accounting for most resting-state signal variance, but it has also been shown to be related to cortical hierarchy, and thus interpreted as a model of heteromodal representation (Mesulam, 1998). The third one, conversely, shows resemblance with the task-positive network (TPN) by Fox et al. (2005a). The two PG maps are shown in Supplementary Fig. S14. A series of Behavioral Analyses (see Supplementary Materials for methods) confirmed that the whole-brain PG1 is anchored at one side to regions with primary functions and at the other to associative areas. The same method illustrates a sort of bimodal distribution of PG3 heteromodality, with DMN areas, at the lower part of the scale, associated with social cognition and emotions, and TPN regions, at the top, related to attention, working memory and reasoning, with primary functions half-way (Supplementary Fig. S14). Thus, PG3 could be used to distinguish between socioaffective and executive associative areas.

The correlations between our four ICs and these two PGs is presented in Table 3. Most correlation approaches 0, except for the one between IC1 and PG3 (*r* = 0.20), and those between IC4 and PG1 and PG3 (*r* = 0.18 and *r* = − 0.16, respectively). According to Cohen (1992), *r* = 0.10 is to be considered a small effect size, while a medium effect size equates to *r* = 0.30. This suggests that resting-state variance is not able to recapitulate brain activity fully.

**Table 3:** Pearson’s correlation between the brain voxels of the four independent components (IC) and the two principal gradients (PG) by Margulies et al. (2016), and percentages of the positive voxels of the ICs overlapping with the positive voxels of the PGs

	Pearson’s *r*		Overlaps (positive voxels only)		
	PG1	PG3	PG1	PG3	PG1 ∪ PG3
IC1	0.04	0.20	55%	65%	83%
IC2	0.08	− 0.03	52%	35%	66%
IC3	0.04	− 0.05	50%	36%	70%
IC4	0.18	− 0.16	73%	12%	76%

**Fig. 5:**
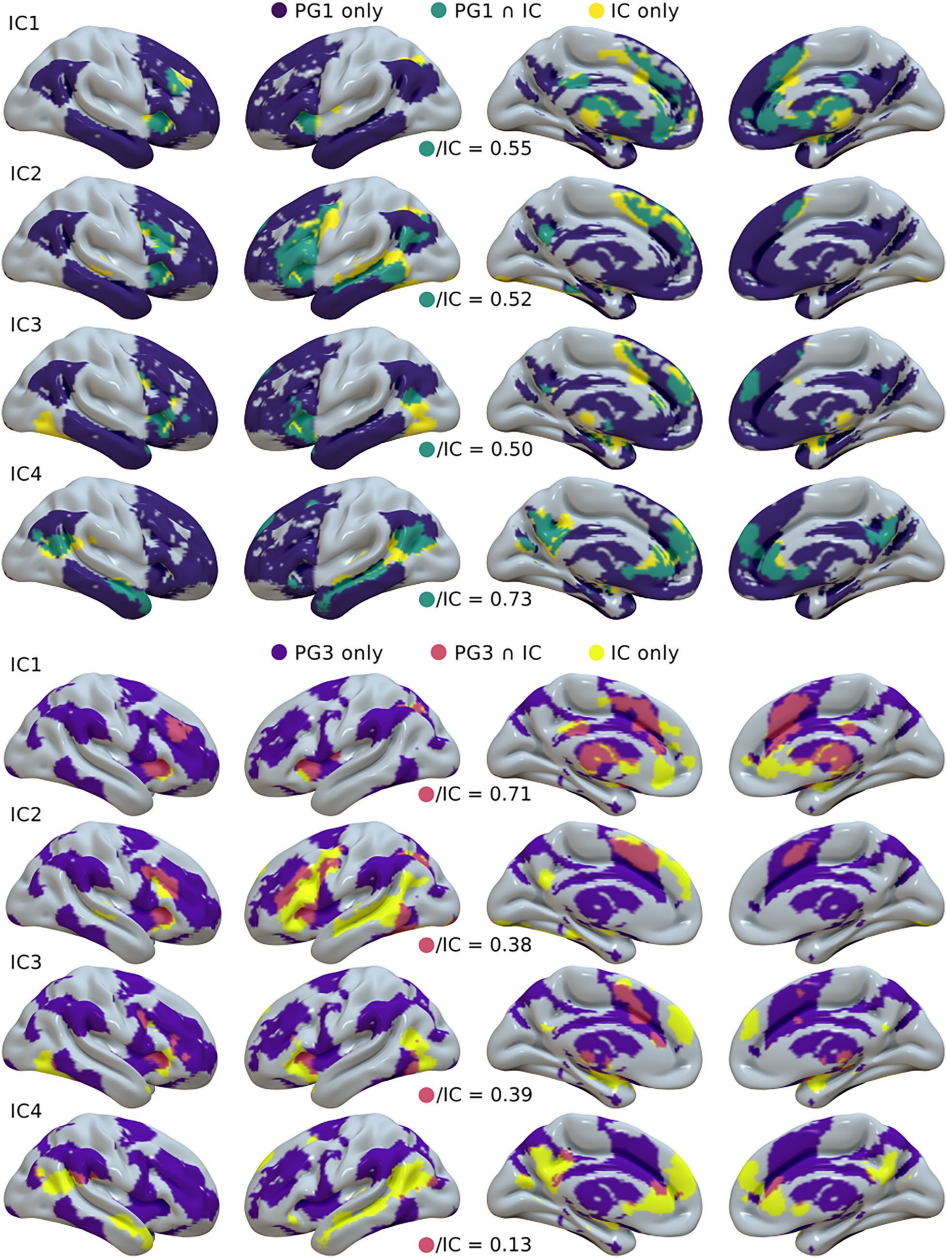
Surface mapping of the overlaps between the volumes of positive voxels of the four ICs and PG1 and 3 by Margulies et al. (2016). For each comparison, the ratio of positive IC voxels overlapping with the positive PG is reported

However, a series of two-sample *t* tests indicates that the average gradient value of positive IC voxels was significantly higher than that of the rest of the brain (*p* ≈ 0 for all ICs and with both PGs). Figure 5 illustrates the overlaps between the positive voxels of the four IC and the two PGs. The percentage of positive voxels of each IC overlapping with those of the two PGs is reported in Table 3. All ICs overlap with at least 50% of their positive voxels with heteromodal regions as defined by PG1, and IC1 also shows high convergence with PG3.

The comparisons of PGs with ALE and PC maps produced similar results: low correlations and high overlaps. All the two-sample *t* tests were highly significant. Correlations and percentages of overlap are shown in Supplementary Table S4. Lastly, we tested if the correlations between ALE maps and PGs could, in turn, be correlated with their placement on the MDS *x* axis, that is, if the alignment between an ALE map and a PG could be predictive of its placement on our internal–external gradient. MDS *x* coordinates correlate strongly and significantly with PG3 (*r* = − 0.69; *p* = 0.02, one-tailed t-test), but not so much with PG1 (*r* = 0.43; *p* = 0.12). This means that the internal–external axis is mostly orthogonal with heteromodality, but is related to a socioaffective-executive gradient.

## Discussion

The present work provides compelling evidence that regions of the DMN are engaged in several tasks, not restricted to those conventionally associated with the resting state and the mind-wandering, and also including semantic reasoning and reward mechanisms. In light of the resulting ALE maps, it is important to bear in mind that all these paradigms were found to activate at least two out of three DMN masks more than chance. Thus, the density of their foci in literature is particularly high within the DMN. However, the density in other RSNs might be just as high as in the DMN. Furthermore, there was no reason to always find meta-analytic activations in the central hubs of the network. In fact, while most maps show at least a cluster in the PCC of mPFC, Imagined Objects/Scenes and Deception seem to involve only areas such as IFG, the precuneus, AG. Conversely, many maps include non-DMN areas associated with task-positive networks such as the SN and frontoparietal network (FPN). This means that there are tasks involving nodes of the DMN along with those included in other networks, thus forming patterns of activation that cross the boundaries of canonical RSNs.

It has been argued that to assess its function, a network should be assessed considering it a spatially and temporally coherent system as a whole, and not in terms of its constituting regions (Jackson et al. 2019). Following this approach, Jackson et al. (2019) reported that the coherent DMN is not involved in semantic cognition, concluding that the DMN and SemN are two distinct networks. However, such consideration could be extended to other cognitive domains, leading to the paradox that no function should be associated to the DMN. In fact, our results indicate that DMN task-related activations span across RSNs, suggesting that canonical networks might have limited heuristic value to understand brain functioning when involved in extrinsic processing. Therefore, task engagement may induce the cooperation between areas belonging to different canonical RSNs. Although relying on intrinsic brain topology, such recruitment would not be strictly constrained by it (Cole et al. 2014; Krienen et al. 2014). Thus, it might involve a flexible shift in brain hubness (Cole et al. 2013; Fransson and Thompson 2020) and a remodulation of cooperative and competitive long-range connectivity patterns (Fornito et al. 2012; Piccoli et al. 2015; Dixon et al. 2017).

There are several obvious reasons explaining why canonical RSNs are a simplistic representation of brain complexity (Pessoa 2014). For instance, they partition the gray matter into non-overlapping volumes, even if in the brain, as in most real-world networks, a node is usually connected to more than one community (Palla et al. 2005; Ferrarini et al. 2009; Yeo et al. 2014; Najafi et al. 2016). In addition, FC changes from rest to task (Arbabshirani et al. 2013; Mennes et al. 2013; Spreng et al. 2013; Cole et al. 2014; Goparaju et al. 2014; Vatansever et al. 2015a, b; Krieger-Redwood et al. 2016; Najafi et al. 2016; Bolt et al. 2017; Moraschi et al. 2020) and dynamically fluctuates over time (Chang and Glover 2010; Hutchison et al. 2013; Calhoun et al. 2014; Preti et al. 2017). The non-stationarity of FC indicates a fluid node recruitment by whole-brain connectivity modules, resulting in time-varying networks. In this regard, de Pasquale et al. (2012) found the DMN to be the system most connected with extra-network regions during epochs of strong internal correlation. More in general, several dynamic FC studies (Chang and Glover 2010; Kiviniemi et al. 2011; Liu and Duyn 2013; Karahanoğlu and Van De Ville 2015) portrayed the DMN as a moving landscape, with a changing spatial distribution and whole-brain correlations over time.

Thus, it is not puzzling that our maps did not reproduce the resting-state DMN. Still, it is interesting to notice that not even TID or the functions more canonically associated with the network are able to show a strong affinity with the DMN masks (Supplementary Table S1 and S4, and Fig. S5 and S6). The lack of a perfect match between the phenomenon of activations (and deactivations) and FC suggests that the resting-state connectivity, while usually regarded as *intrinsic* (Fox et al. 2007; Vincent et al. 2007; Van Dijk et al. 2010; Cole et al. 2014, 2016; Tavor et al. 2016), can inform us only partially about brain functioning. This reasoning does not invoke a complete abandonment of RSNs, as it can be argued that they maintain a sort of taxonomic utility, in the sense that they are well-known structures that help to categorize brain anatomy and topology (Uddin et al. 2019). In this regard, it makes sense to investigate the functions of the resting-state DMN. For instance, it may be reasonable to conclude that the canonical DMN could be seen as an entity having the role of integrating different cognitive aspects thanks to the interactions between its subcomponents (Wen et al. 2020). This is in line with the observation that the DMN displays a diversified cytoarchitecture and connectivity with the rest of the brain (Paquola et al. 2021). It is also consistent with its strong dynamical connectivity with external nodes, including task-positive regions (de Pasquale et al. 2012; Karahanoğlu and Van De Ville 2015), which might explain their involvement in our maps. These considerations also resonate with the theoretical proposals that the function of DMN might be that of reducing the entropy or the free energy of the brain (Carhart-Harris and Friston 2010; Carhart-Harris et al. 2014), or that of a sense-making network integrating new information with past knowledge (Yeshurun et al. 2021), in agreement with its dense connectivity and its topologically central position in the connectome (van den Heuvel and Sporns 2011; Tomasi and Volkow 2011). In any case, besides any possible interpretation, our findings highlight that the tasks that significantly activate the canonical DMN cannot be simply reduced to it, and that they show anatomical and functional diversity between each other.

Although these tasks may be easily linked to certain internal forms of mentation, the nature of some of them highlight that such internal cognition seems to be crucial for external tasks. It is important to point out that since we tested the DMN for significant paradigms, the resulting ALE maps represent the activations of a specific set of experimental tasks, defined in an operational way. On the contrary, psychological definitions such as those implemented by the Behavioral Analysis (Lancaster et al. 2012) span across paradigms. For instance, the behavioral domain of Semantics entails Covert Word Generation, Self-Reflection, Encoding, Passive Listening, Visuospatial Attention, and other tasks. These range from the most extrinsic and active functions to forms of internal cognition that may be involved in mind-wandering. Therefore, working at the level of paradigms allowed us to focus on the kind of activities the DMN regions are engaged in.

For instance, we found Reward paradigms to be associated with canonical DMN. This is mostly due to a large amount of foci covering the whole mPFC. The latter is known to modulate reward mechanisms (Ferenczi et al. 2016), to respond to the outcome of risky decisions (Xue et al. 2009), and to activate when receiving a social reward (Martins et al. 2021). Reward mechanisms could be considered as an example of functions meant to monitor internal states, yet crucial for the implementation of behavior. As a matter of fact, reward dynamics involve the perception of somatic states and their emotional and self-related processing, which implies the activity of mPFC, ACC, SMA, along with BG, amygdala, and insula (Verdejo-García and Bechara 2009; but see also Dunn et al. 2006). At the same time, reward functions are critical for learning, risk-taking, and behavior in general (Schultz 2015). Furthermore, as an example of collaboration between internal and external cognition, the reward system is functionally connected to the DMN during mental simulation of the outcome of goal-directed behavior (Gerlach et al. 2014).

Deception is another paradigm class arguably standing in between internal and external cognition. Deceiving someone requires social cognition, which is typically associated with the DMN internal mentation (Buckner et al. 2008). However, it might be argued that deceiving is a more external activity than ToM. While the latter only requires to represent other people’s mental states, the former also implies goal-directed programming of one’s own behavior to successfully deceive the other (Lisofsky et al. 2014). Moreover, specific attentional and executive functions are likely to be necessary to perform a deception paradigm (Christ et al. 2009; Farah et al. 2014). As a matter of fact, ToM appeared close to TID on the first MDS axis and Deception was at the extreme opposite side.

In general, the MDS results suggested that the DMN-related experimental paradigms and their associated activation maps could be arranged along an internal–external, midline-lateral axis. This observation is consistent with the growing body of evidence pointing out that the DMN is recruited during task execution (Crittenden et al. 2015; Vatansever et al. 2017; Murphy et al. 2018), and suggests that its function may be related to some form of high-level cognition, detached from the here and now, but still crucial for goal-directed behavior (Konishi et al. 2015; Benedek et al. 2016). At the same time, our results also clearly illustrate that when engaged in external operations, the network activations shift from the spatial representation typical of the rest condition. In fact, the ALE maps associated with more extrinsic paradigms display a clear dissimilarity from the canonical representation of the DMN. Specifically, they involve peripheral nodes of the system such as AG and IFG, they often show weak or no activation at all within the midline core, and they sometimes engage SN and FPN considered to be anticorrelated to the DMN at rest (Fox et al. 2005a).

To summarize the anatomical variance of such extrinsic and intrinsic brain activity, we performed a PCA and an ICA. A large part of the task-related variance is explained in the form of proactive modes of internal cognition (PC1, Reward; PC2, Semantic Monitor/Discrimination), in opposition to core areas active during rest and social cognition paradigms (PC4). The four-component ICA replicated the PCA results closely, with the meaningful difference that the first two components were found to be anticorrelated with regions associated with Emotion Induction. Therefore, affective functions may constitute an important factor of DMN reorganization during task execution. We also note that PC2 and IC2 (but PC1 as well) resemble the SemN (Noonan et al. 2013; Chiou et al. 2020; Evans et al. 2020; Milton et al. 2021). Furthermore, they remind the transitional module serving an integrative function with FPN observed by Fornito et al. (2012), as well as with the Overlapping Community six found by Najafi et al. (2016). Consequently, we suspect that they both serve some form of integration between the DMN core and the task-positive areas for the execution of more external tasks.

It has been proposed that the DMN topography might be crucial to understand its function: its placement at the furthest distance from areas with primary functions could imply that it carries out the most abstract representations and that it integrates the broadest range of information (Smallwood et al. 2021). According to this, it could be expected that our ICs of DMN activity were somewhat related to PG1 by Margulies et al. (2016), which is associated with the topography of cortical hierarchy while explaining most of the resting-state variance. However, none of our ICs (or ALE maps) showed even a mediocre correlation with PG1, and, most importantly, the similarity of ALE maps with PG1 was not correlated with the first MDS axis. This does not imply that PGs (or, once again, resting-state FC in general) are not relevant for the understanding of brain activity. On the basis of the existing literature (Smith et al. 2009; Laird et al. 2013), we would expect that if data reduction techniques were applied to a wider meta-analytic repertoire of functions, the resulting components would match the resting-state PGs much better. However, it points out that the DMN-to-primary areas PG1 is not sufficient to predict DMN active modes, but just to identify the associative regions that such modes are more likely to load. Therefore, we might be in need of more detailed models to understand brain dynamical reorganizations. In line with this, recent work by Paquola et al. (2021) reports that DMN connectivity is aligned to a cytoarchitectural axis rather than with PG1. Interestingly, the similarity between our ALE maps and the TPN-to-DMN PG3 correlates with our external-to-internal MDS axis, suggesting that a socioaffective-executive differentiation between heteromodal regions might play a role in explaining DMN functional rearrangements.

The absence of the ventromedial PFC (vmPFC) from our results deserves to be briefly commented. The only difference between our TID ALE map and that by Laird et al. (2009) is that our mPFC cluster does not include the most ventral areas of the region. Of the other maps, only Reward involves vmPFC. IC1 (and PC1) includes the vmPFC, but IC4 (and PC4), which loads on TID and ToM, does not. This is a bit surprising, as the vmPFC is considered part of the DMN, and it is present in the ROIs utilized for the Paradigm Analysis (Supplementary Fig. S1). A possible explanation might be related to an attenuation of the detected effects in this area by the studies included in the meta-analysis, as the result of strategies having the purpose to minimize artifacts, which are common in the most ventral parts of the brain. However, it would be unclear how this could explain the difference between our findings and those by Laird and colleagues. The difference may be explained by our larger database and updated ALE algorithms (Eickhoff et al. 2016, 2017). If the vmPFC absence from our maps was justified, it would be possible that it would be another difference between activations and connectivity.

An unexpected finding was that our meta-analysis was powerful enough to produce juxtapositions of components that were reminiscent of the works by Braga and colleagues (Braga and Buckner 2017; Braga et al. 2019; DiNicola et al. 2020). As their results were originally obtained with minimally smoothed individual data, it is remarkable that something similar was achieved by our method. Another recent meta-analysis (Ngo et al. 2019) obtained a similar result, decomposing the inter-experiment DMN variability in two components. However, our methodology was able to highlight sharp contrasts between neighboring areas just analyzing the final ALE maps. This is particularly evident for PC3 and IC3, both related to Emotion Induction. In both components, the mPFC is parcellated in alternated bands of network and anti-network, with SMA and anterior dmPFC positively associated with the paradigm, and posterior dmPFC and central mPFC showing negative values. The opposite pattern was shown by IC1, related to Reward. The PCC was tightly segmented as well, particularly in IC2 and IC3, where a small section of positive voxels was surrounded by negative values. More in general, PC2, PC3, IC2, and IC3 indicated a preferential engagement of a more posterior portion of PCC in semantic monitoring and induction of emotions, with negative scores in a more anterior part. On the contrary, reward mechanisms showed the opposite pattern in IC1 and, to some extent, in PC1. Such rostro-caudal segmentation of the PCC was also observed by Leech and colleagues during the execution of an attentional task, with the caudal portion displaying less integration with the DMN and less segregation with the task-positive regions (Leech et al. 2011). To summarize, the midline core, clearly associated with TID (PC4 and IC4), appeared much more jagged in other components, presenting patterns of correlation and anticorrelation in a gradient around the corpus callosum.

## Limitations and future directions

The main limitation of this work derived from the choice of using the BrainMap database as the only source of activation foci. This was done for the sake of consistency, as the tasks significantly associated with the DMN were found using the Paradigm Analysis, which operates on the BrainMap database. Obviously, we could have integrated our data with experiments found through a systematic search on PubMed. However, a larger dataset could have possibly translated into ALE maps in disagreement with the Paradigm Analysis results, for instance without any significant activation within DMN nodes. Importantly, this would have not been necessarily due to a better representativity of the larger database, but possibly just because of a different coding of the paradigms. Having to choose between internal consistency and a larger sample size, and considering the amplitude of the functional BrainMap data archive (more than 18,000 experiments in total), we preferred to conduct our whole research within the same database. This choice returned an underpowered Self-Reflection ALE. However, by removing it from the data, we obtained a similar MDS and identical PCA and ICA results. Moreover, an exploratory ALE using a liberal uncorrected threshold with *p* = 0.001 (not shown) revealed additional clusters in the dmPFC, mPFC, dlPFC, left insula, and IFG. A similar map would be rather consistent with our general results. Therefore, a more representative Self-Reflection map would probably be more heavily loaded by those components representing the DMN internal modes of cognition such as PC4 and IC4, rather than leading to radically different findings.

Our results indicate that several co-activation networks converge on the resting-state DMN nodes. For instance, the Semantic Monitor/Discrimination ALE map seems to indicate that the hubness of the DMN has been moved from the midline core to the left IFG and middle temporal gyrus, which are peripheral nodes during rest, and to left insula and SMA, these latter parts of the SN. This evidence suggests that during task execution the nodes of the DMN could update their FC and dynamically modify their topological centrality, as observed by Cole et al. (2013) for the FPN. However, the present work did not directly test this hypothesis. In particular, during semantic monitoring tasks, left IFG could be coupled with the middle temporal cortex, and insula with SMA, forming two relatively independent modules. Alternatively, they could be all reciprocally co-activated in a rich-club fashion. The methods used in the present research cannot disambiguate between these and other possible hypotheses. Thus, future works could be addressed towards the implementation of some methods to estimate networks of co-activations from meta-analytical data (Toro et al. 2008; De La Vega et al. 2016; Mancuso et al. 2019; Cauda et al. 2020) so as to assess the centrality of the nodes across tasks. Task-based stationary or dynamic FC could be used as well.

The present study raises a compelling question concerning the mechanism arranging the dynamical shifts from the midline core during task. An influential model proposed that the anterior insula could be responsible for coordinating the interplay between the DMN and frontoparietal task-positive regions (Sridharan et al. 2008; Menon and Uddin 2010). The insula was actually found by Najafi et al. (2016) to be connected to several modules despite a relatively low degree centrality, both during rest and emotional tasks. The AG was implicated in the same role and identified, by Kernbach et al. (2018), as the mediator of the interplay between different RSNs. Alternatively, the amPFC was shown to be activated during switches between stimulus-independent and stimulus-oriented thoughts (Gilbert et al. 2005), suggesting to play a role in the coordination of internal and external modes of mentation. Future studies could further investigate the issue to clarify which areas or mechanisms are involved in the task-based DMN rearrangements.

## Conclusions

The present study indicates a series of tasks activating the DMN that are not exactly internal, nor completely external. These activations involve DMN regions but also large parts of other canonical RSNs, in particular SN and FPN. Furthermore, they appear to be arranged in an anatomo-psychological gradient starting from the most internal functions, which activate the midline core, towards such relatively extrinsic mode of brain function, which involves the lateral cortices. In the light of our results, such extrinsic mode is especially related to reward, semantic, and emotional functions.

Ultimately, our findings highlight that resting-state scaffoldings do not suffice to explain the task-related anatomical variance of the active brain, which displays a much richer functional diversity, and shows more spatial complexity than it could be expected just observing intrinsic connectivity.

## Data and code availability of data and material

Data used in this meta-analysis were obtained from BrainMap (http://brainmap.org/), a publicly available database. Analyses were performed with open source software. The results are available at https://github.com/SCCabanillas/DMN-task-based-modulation.

## Supplementary Information

Below is the link to the electronic supplementary material.

Supplementary file1 (PDF 2873 KB)
