# Supplementary material for: Tasks activating the default mode network map multiple functional systems
Source: Brain Struct Funct. 2022 Feb 18;227(5):1711–34. doi: 10.1007/s00429-022-02467-0 (PMC9098625; doi:10.1007/s00429-022-02467-0)
Supplement: Supplementary file 1 — Supplementary file1 (PDF 2873 KB) [file 429_2022_2467_MOESM1_ESM.pdf]

Tasks activating the Default Mode Network map multiple functional systems

Supplementary online materials

Lorenzo Mancuso[1], Sara Cavuoti-Cabanillas[2], Donato Liloia[1,3], Jordi Manuello[1,3], Giulia Buzi[1], Franco Cauda[1,3], Tommaso Costa[1,3]

[1] FOCUS Lab Department of Psychology, University of Turin, Turin, Italy.

[2] Department of Physics, University of Turin, Turin, Italy.

[3] GCS-fMRI, Koelliker Hospital and Department of Psychology, University of Turin, Turin, Italy.

Figure S1: Surface mapping of the three DMN masks used, their union, their intersection, and the result of selecting the voxels shared by at least two masks.

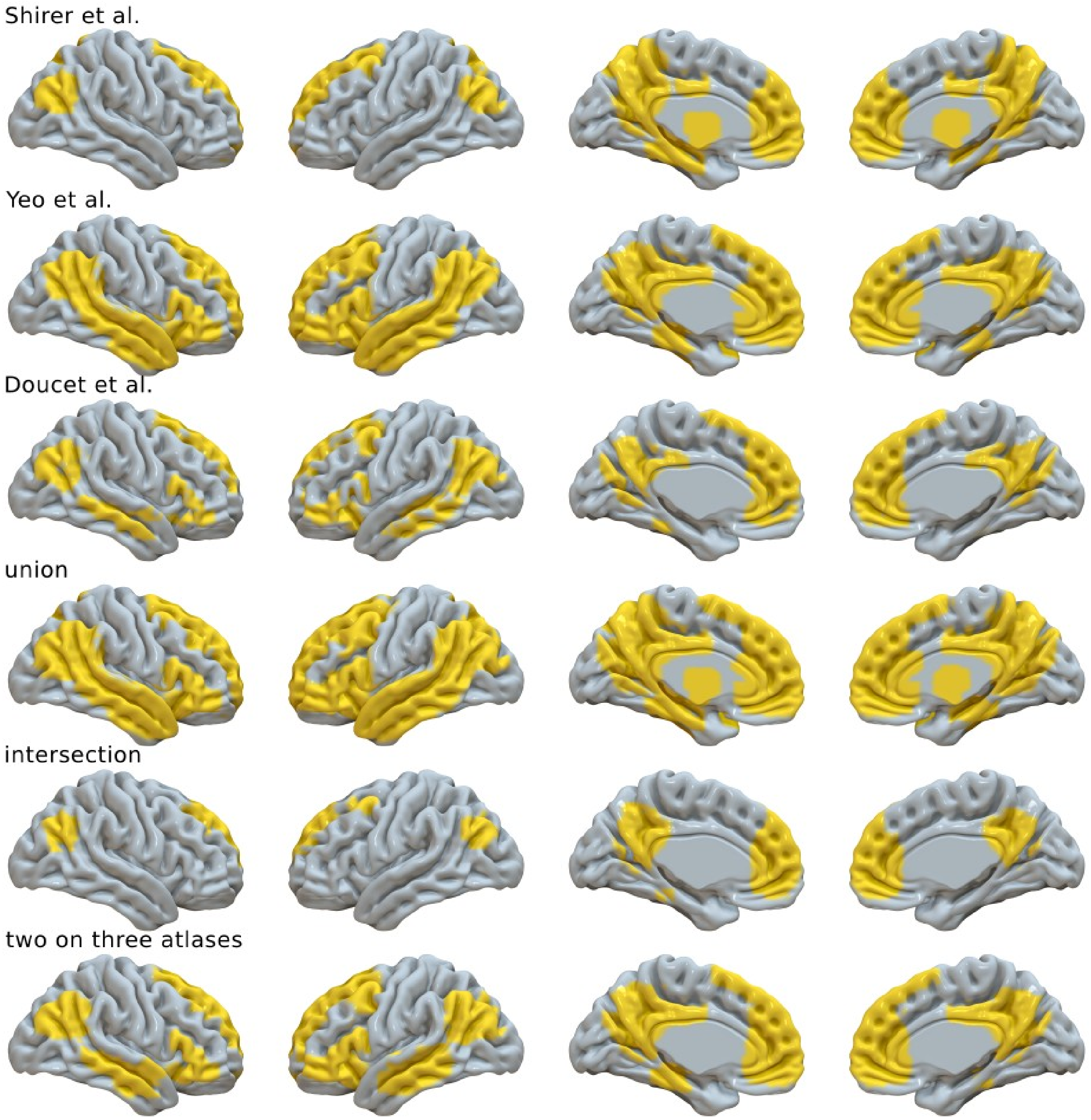

Figure S2: PRISMA flow chart of the selection of studies.

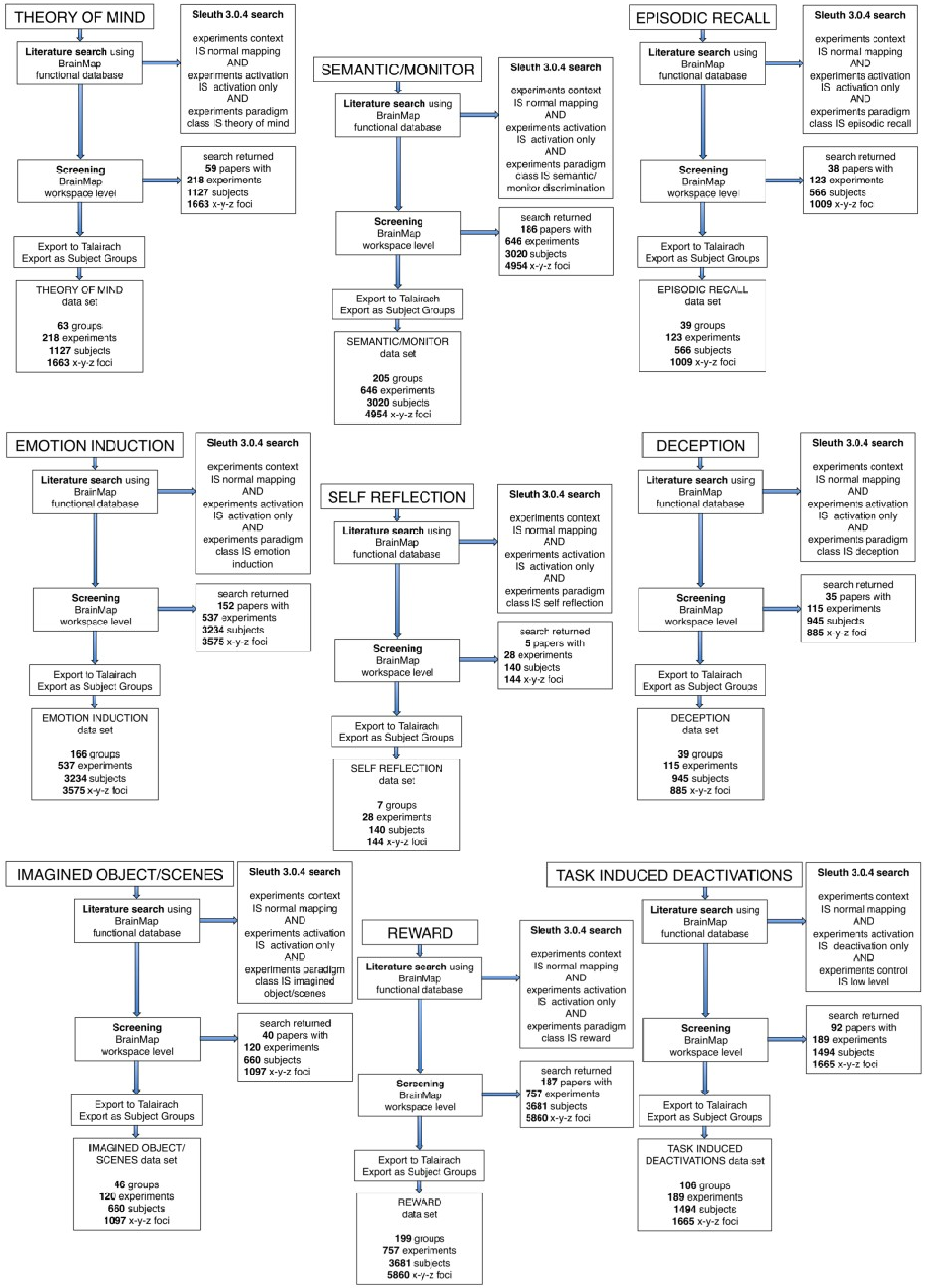

Figure S3:Surface mapping of the Activation Likelihood Estimation maps obtained with the Fail-safe procedure with 6% of file-drawer effect.

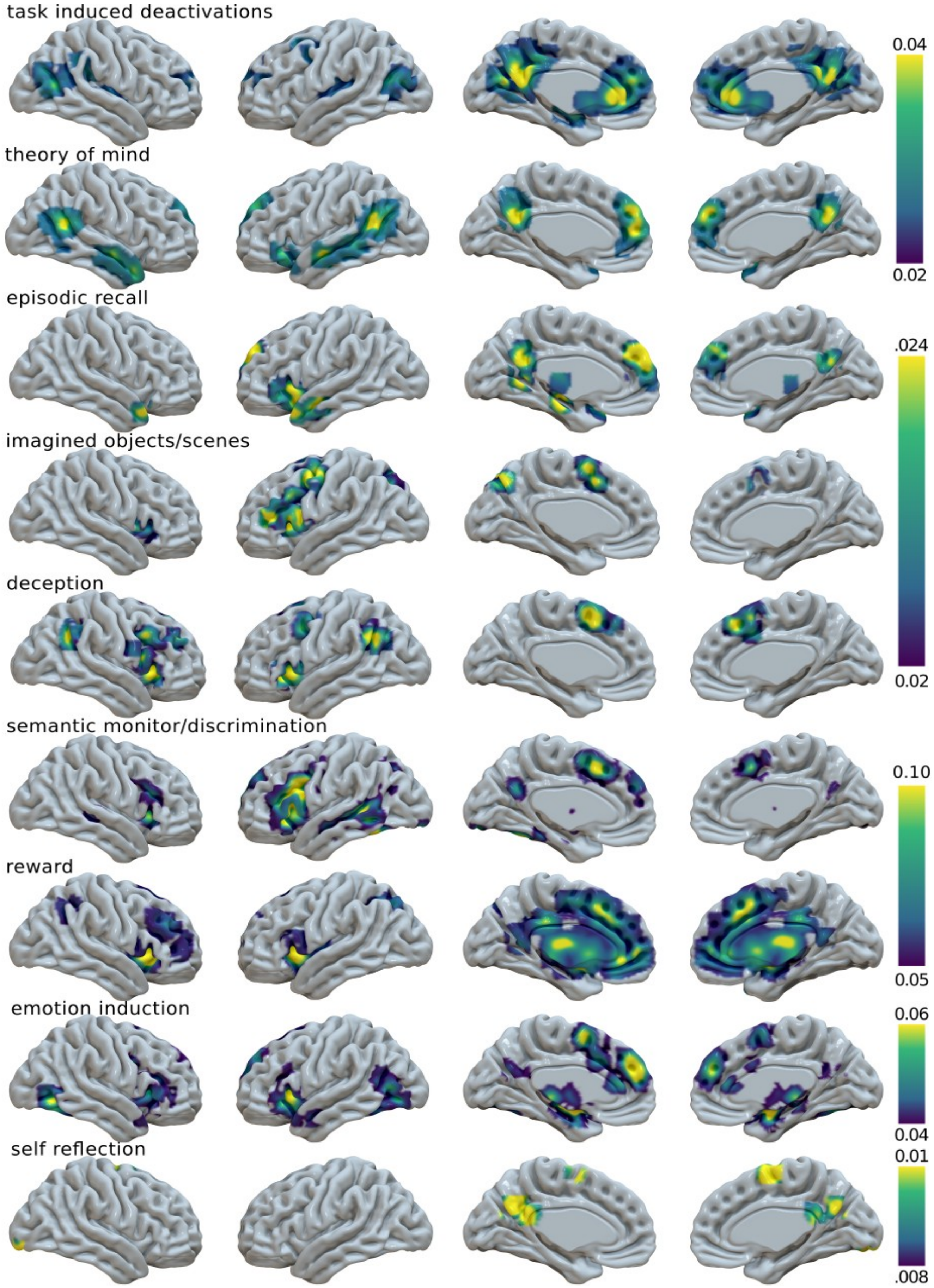

Figure S4: Surface mapping of the Activation Likelihood Estimation maps obtained with the Fail-safe procedure with 60% of file-drawer effect.

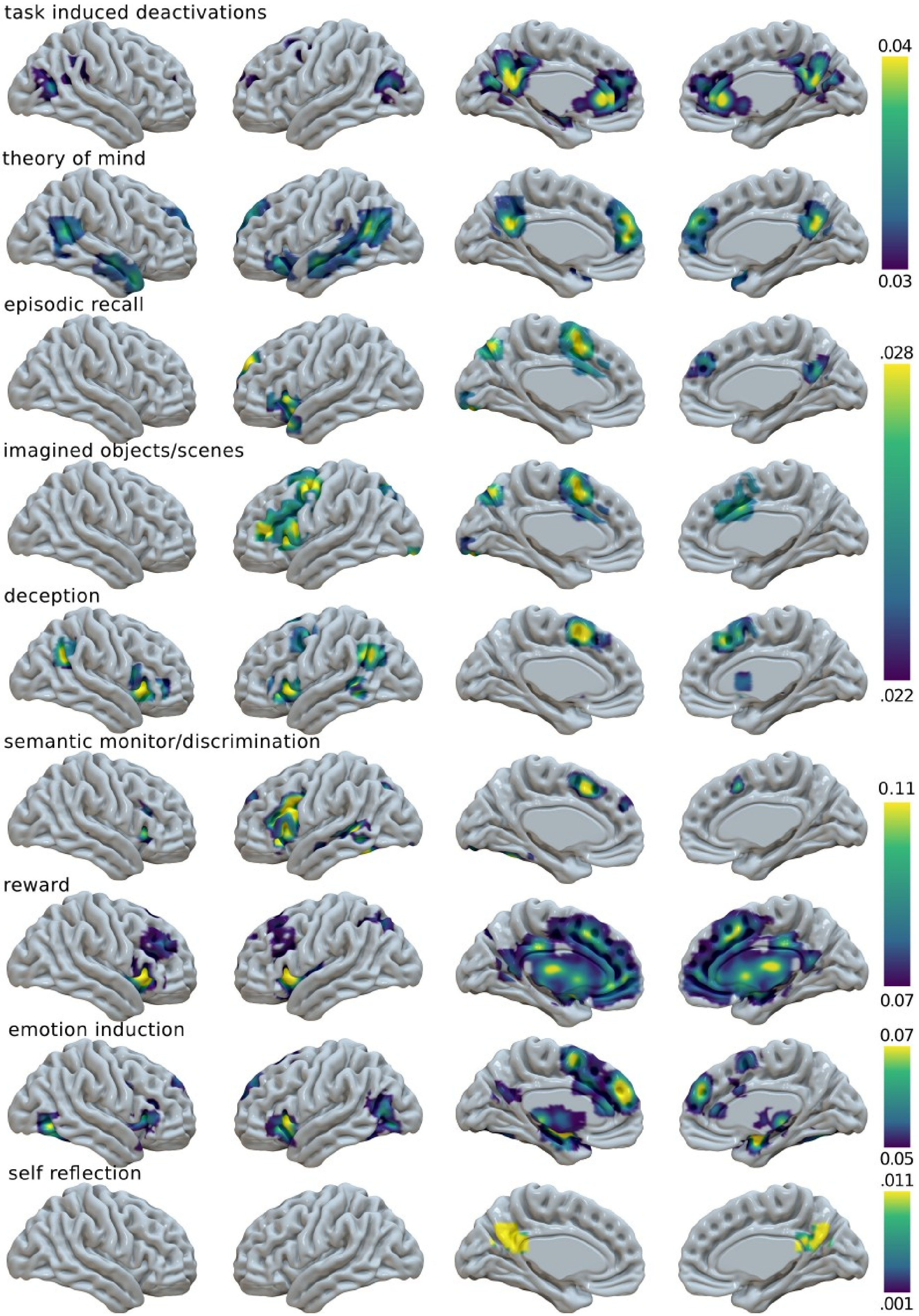

Figure S5: Volume mapping of the overlaps between the ALE maps and the three DMN masks used. First three columns cut: x = -2, y = 14, z = 8. Fourth column x cut: TID = -41; episodic recall, deception = -46; reward = -38; self reflection = 6; others = -48.

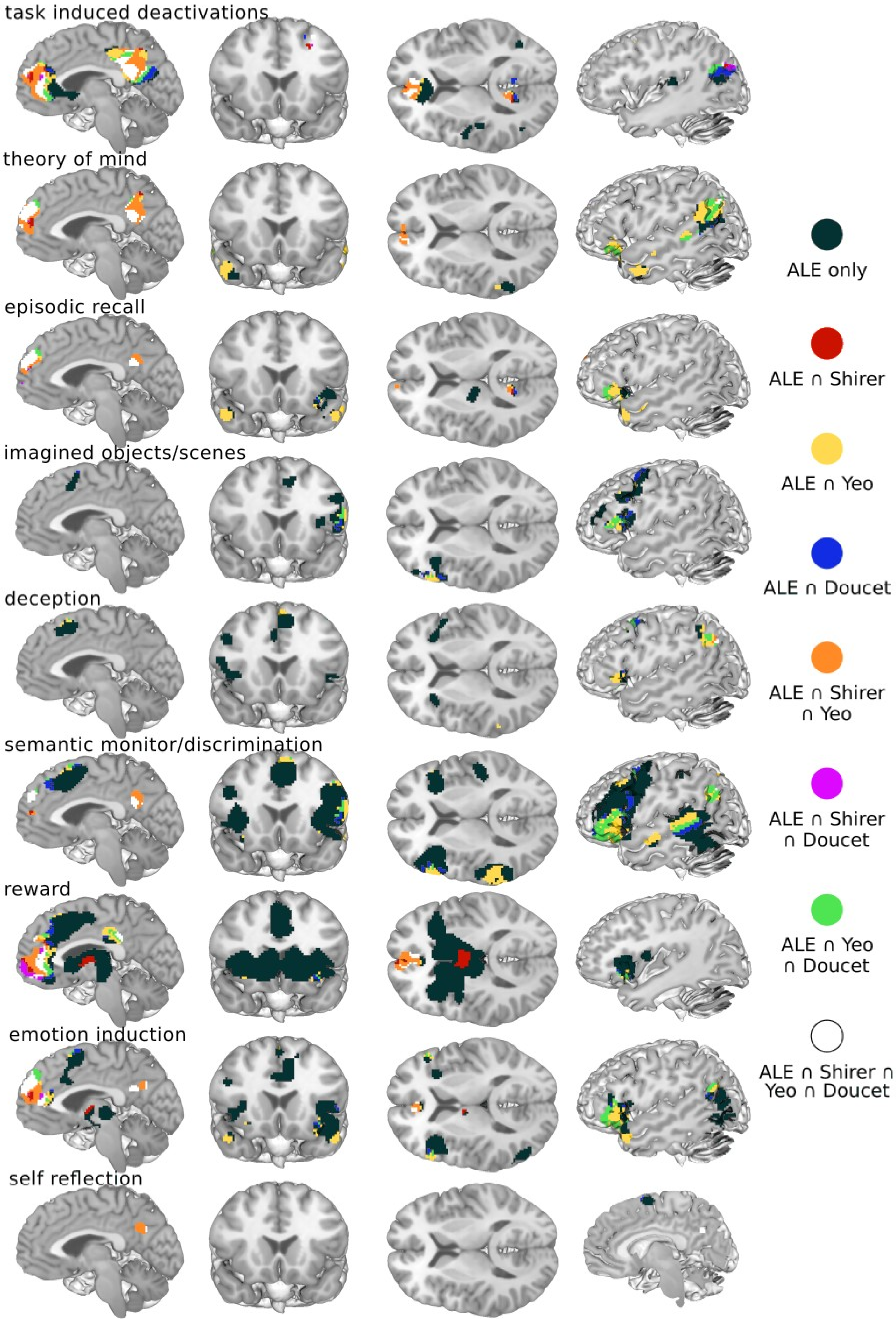

Table S1: Jaccard index values for each paradigm and each atlas. In Shirer et al., just as dorsal and ventral DMN masks were merged into one (see Methods), the same was done for anterior and posterior salience, high and primary visual, and left and right ECN. Please note that the network names change between the three parcellations. The Ventral Attention network by Yeo et al. corresponds to the Salience network of others. ECN, CEN and frontoparietal network are more or less the same network. The language network by Shirer et al. includes several areas that are part of the DMN in the other two parcellations.

	Deception	Emotion Induction	Episodic Recall	Imagined Objects-Scenes	Reward	Self Reflection	Semantic Monitor-Disc.	TID	ToM
Shirer et al.									
DMN	0	0.06	0.05	0	0.06	0.01	0.03	0.17	0.12
Auditory	0	0	0	0	0	0	0.01	0.01	0
Basal Ganglia	0	0.03	0.01	0	0.08	0	0	0	0
ECN	0.04	0	0	0	0.02	0	0.03	0.01	0.02
Language	0.04	0.04	0.02	0.02	0	0	0.07	0.01	0.15
Precuneus	0	0	0	0.01	0.01	0.01	0.01	0.03	0
Salience	0.08	0.07	0.02	0.03	0.1	0.01	0.06	0.02	0.01
Sensorimotor	0	0	0	0	0	0	0	0	0
Visual	0	0	0	0	0	0.01	0.02	0	0
Visuospatial	0	0.01	0	0.06	0.01	0	0.08	0	0
Yeo et al.									
Visual	0	0.03	0	0	0	0	0.04	0.03	0
Somatomotor	0	0	0	0	0.01	0	0.02	0.01	0
Dorsal Attention	0	0.02	0	0.03	0.01	0	0.06	0.02	0.02
Ventral Attention	0.06	0.07	0.01	0.04	0.09	0	0.09	0	0.01
Limbic	0	0.01	0.01	0	0.01	0	0	0	0.01
Frontoparietal	0.03	0.02	0	0.03	0.05	0	0.09	0.02	0
Default	0.03	0.07	0.04	0.01	0.05	0.01	0.08	0.11	0.15
Doucet et al.									
CEN	0.04	0.03	0	0.02	0.05	0.01	0.03	0.01	0.01
SAL	0.04	0.03	0	0.05	0.04	0	0.08	0.01	0
SMN-AUD	0	0	0	0	0	0	0.02	0.01	0
DMN	0.04	0.06	0.05	0.02	0.04	0.01	0.07	0.14	0.13
VIS	0	0.02	0	0	0	0	0.02	0	0

Figure S6: Pie charts of the Jaccard index values for each paradigm and each atlas. In Shirer et al., just as dorsal and ventral DMN masks were merged into one (see Methods), the same was done for anterior and posterior salience, high and primary visual, and left and right ECN. Please note that the network names change between the three parcellations. The Ventral Attention network by Yeo et al. corresponds to the Salience network of others. ECN, CEN and frontoparietal network are more or less the same network. The language network by Shirer et al. includes several areas that are part of the DMN in the other two parcellations.

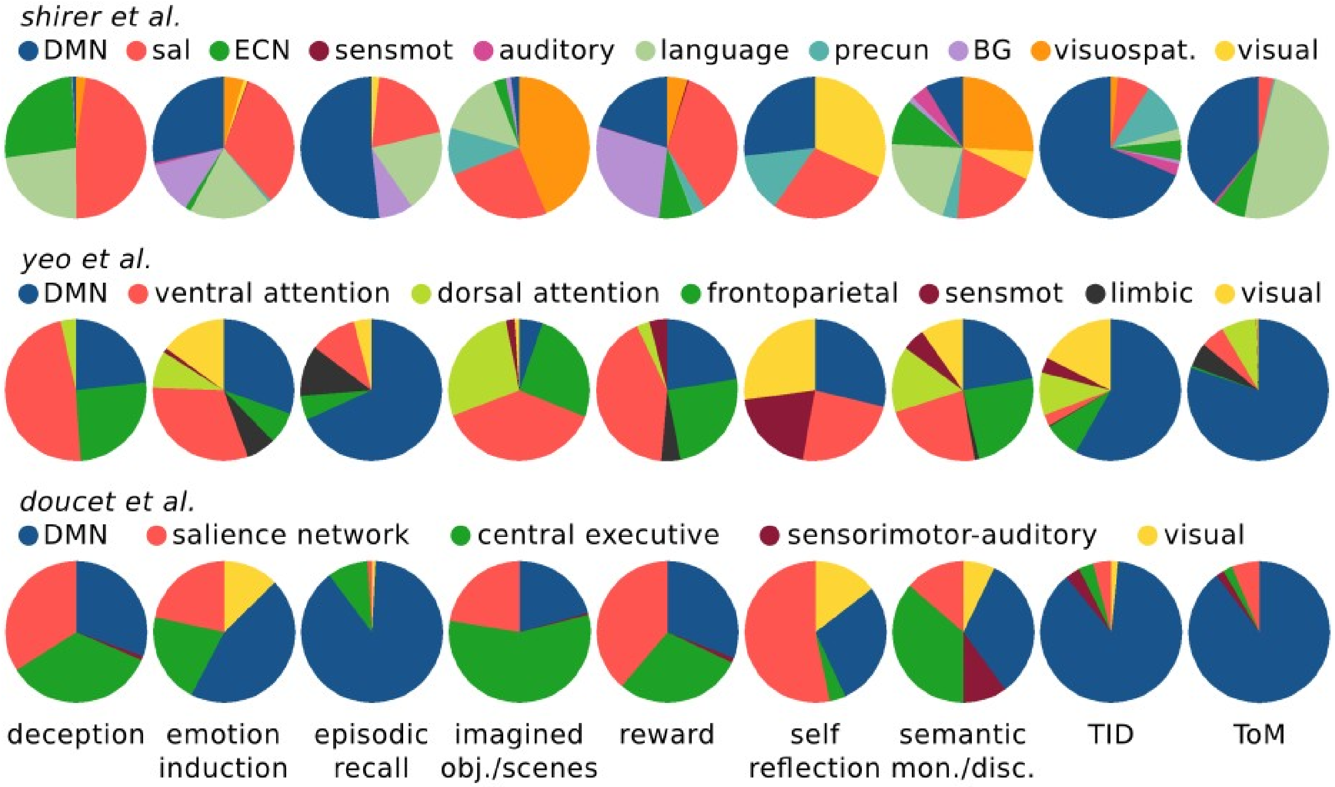

Figure S7:Two-dimensional scaling graph of the main analysis. The volume maps are centered on the MDS coordinates.

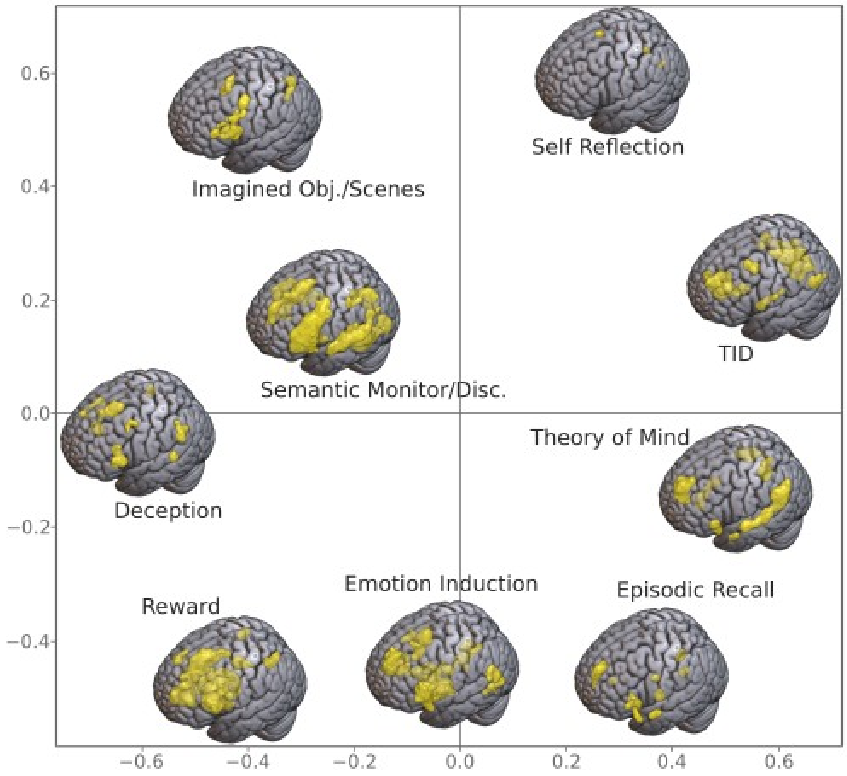

Figure S8: Two-dimensional and three-dimensional scaling graphs calculated excluding the condition Self-Reflection from the analysis.

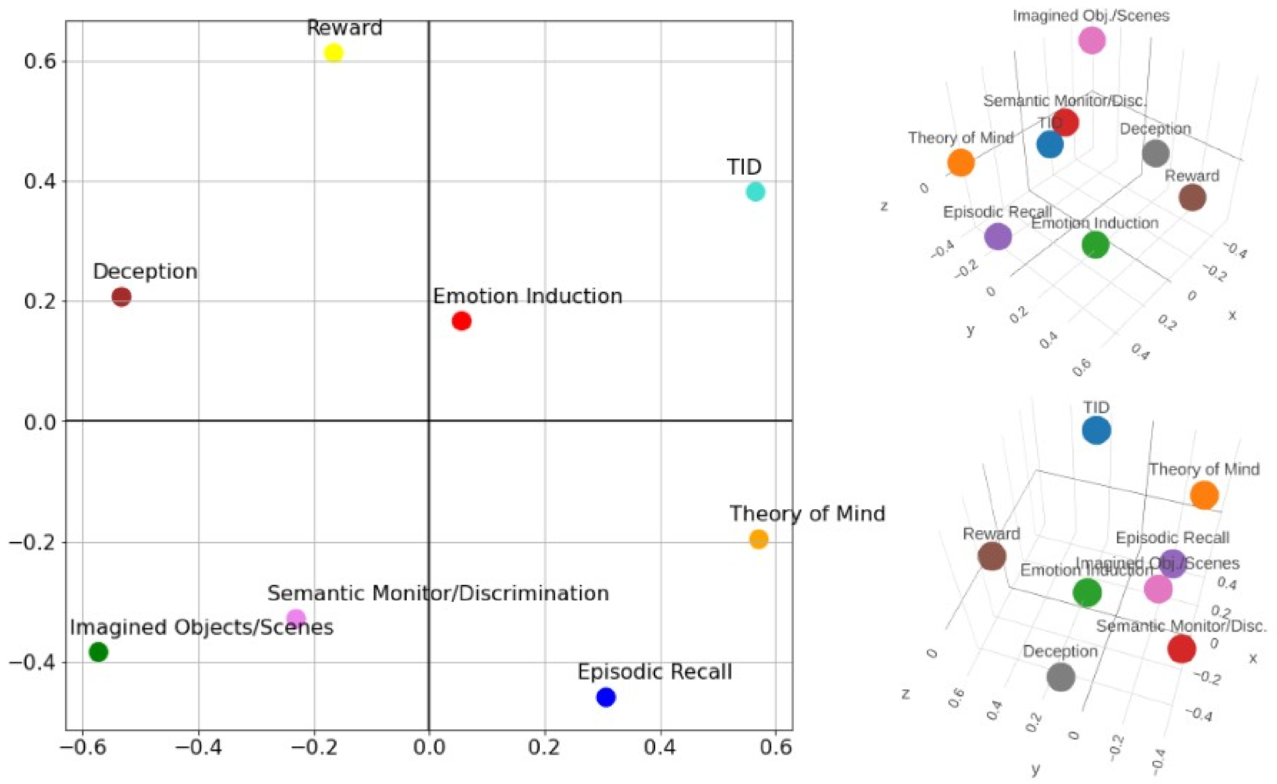

Testing the MDS interpretation

We interpreted the MDS results in terms of a lateral-medial axis and a dorsal-ventral axis. This interpretation was partly justified by the fact that our distances were calculated from the Pearson correlations between the maps, and thus they are informed only by their spatial differences. However, this does not necessarily imply that these differences should be arranged along the major brain axes. Also, although not readily apparent to us, it might be that the third MDS axis could be explained by rostro-caudal activations. We tested these hypotheses with the following procedure. First, we estimated the laterality, dorsality and rostrality of our ALE maps. An index of laterality L (not to be confused with the lateralization index LI [Desmond et al., 1995] that is often incorrectly called laterality index) was calculated as:

$$L = \frac{\sum_{i=1}^{N} (v_i * |x_i|)}{\sum_{i=1}^{N} v_i}$$

where v_i stands for the ALE value of a given voxel i, and x_i is the x coordinate of voxel i, taken in absolute value. Note that this formula is extremely similar to the calculation of the Talairach x coordinate of the image's weighted center of mass. In practice, the value of each voxel is weighted by its x coordinate, and their average (or, simplifying, their sum) is normalized by the average (or sum) of the unweighted map. Taking the x coordinate in absolute value has the effect of weighting the lateral voxel more than the central ones. L could be seen as the distance (in mm) of the weighted center of mass from the midline, if the left hemisphere was flipped over the right one. Similarly, the indices of dorsality and rostrality D and R were computed as:

$$D = \frac{\sum_{i=1}^{N} (v_i * z_i)}{\sum_{i=1}^{N} v_i} \quad , \quad R = \frac{\sum_{i=1}^{N} (v_i * y_i)}{\sum_{i=1}^{N} v_i}$$

where z_i and y_i are the z and y coordinates of voxel i, respectively. These are simply the z and y Talairach coordinates of the weighted center of mass of the map.

Then, we correlated the coordinate assumed by an ALE map on a given 3D MDS axis with the index that corresponded to our hypotheses. We hypothesized that the MDS x coordinates correlated (negatively) with L, and that the MDS y coordinates correlated (positively) with D. We also explored the possibility that the MDS z axis could be correlated with R even if we were not able to see this relation in our results.

As hypothesized, the first MDS axis was correlated with the laterality of the maps with r = -0.6 (p = 0.045, one-tailed t-test). Some observations did not fit our interpretation perfectly. In particular the Theory of Mind map, despite its obvious medial activations (and its longstanding association with the canonical DMN) scored more lateral than expected because of its strong activations in the angular gyrus. Furthermore, Reward was found in the MDS axis closer to the lateral maps despite showing large medial activations, possibly due to its marked insular involvement and its medial activations in non-DMN, salience areas (supplementary motor area). Thus, the placement of the maps on the MDS x axis was mostly consistent with our hypothesis of an anatomical lateral-medial axis, and even its most obvious outlier fit an internal-external psychological description. In fact, Theory of Mind is considered an internal function, while the Salience Network activations of Reward suggest a more external mode of cognition.

Our second hypothesis was that the MDS *y* axis could be explained by a dorsal-ventral arrangement of activations. Indeed, Pearson's correlation between the MDS *y* axis and the dorsality index *D* was $r = 0.93$ ($p < 0.001$). Lastly, we tested if the third MDS axis was correlated with rostrality *R*. As expected, the two variables were not correlated, with $r = 0.06$ ($p = 0.44$). See Table S3 and Figure S6 for further details.

Table S2: Indices of laterality, dorsality and rostrality of each ALE map, and their MDS coordinates on x, y and z axis.

	L	***D***	***R***	***MDS x***	***MDS y***	***MDS z***
Deception	34.75	21.84	1.06	-0.56	-0.05	0.12
Emotion Induction	23.97	4.76	1.4	-0.08	-0.48	0.09
Episodic Recall	21.24	6.82	10.43	0.27	-0.43	-0.27
Imagined Obj./Scenes	31.91	31.33	-1.88	-0.39	0.46	-0.22
Reward	14.54	9.11	8.5	-0.19	-0.09	0.58
Self Reflection	8.98	29.51	-47.06	0.34	0.56	0.01
Semantic Monitor/Disc.	36.68	13	-7.54	-0.32	-0.01	-0.38
TID	12.61	18.91	-17.76	0.48	0.08	0.38
Theory of Mind	31.29	13.84	-13.72	0.46	-0.03	-0.31

Figure S9: Scatter plots of the three MDS axes and the respective center of mass index.

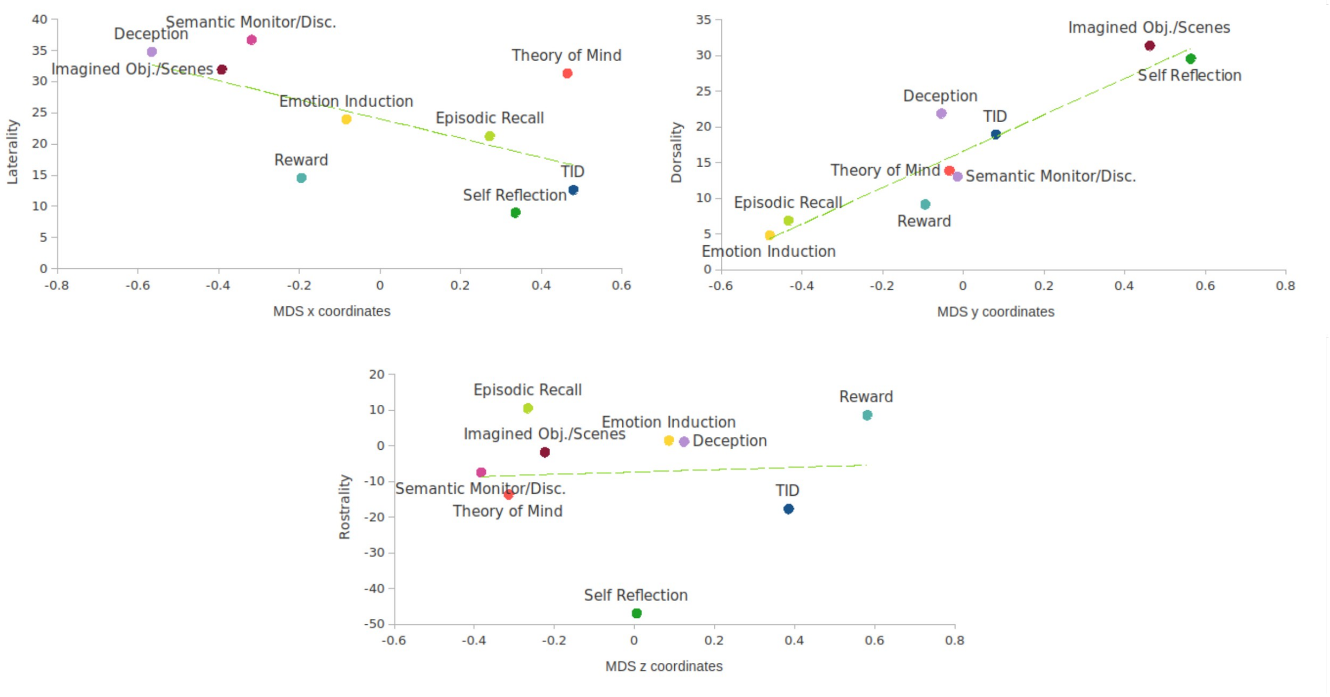

Figure S10: Principal component analysis. Left: surface mapping of the voxel-wise scores. Right: bar graphs of the component loadings for each map.

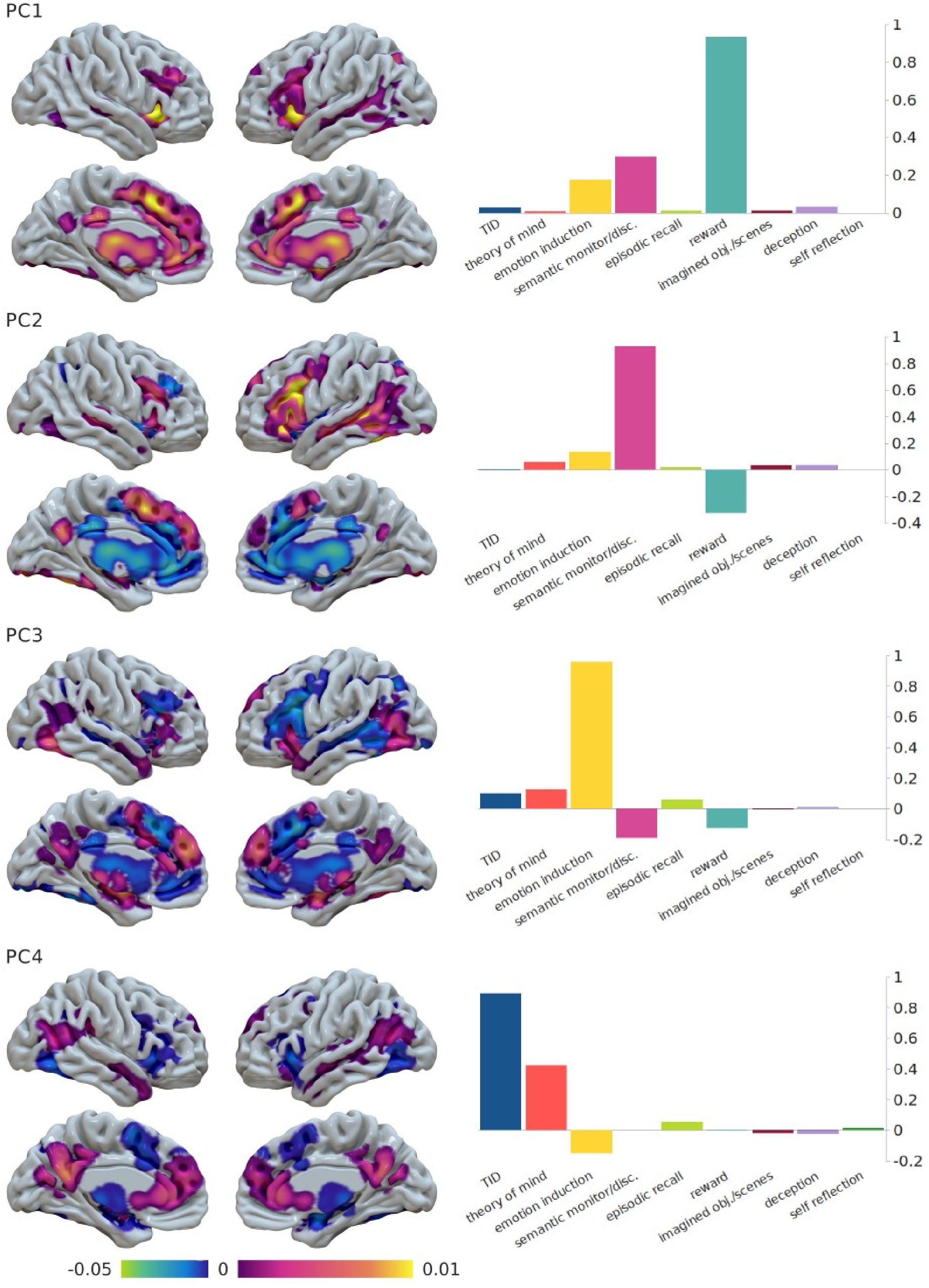

Table S3: Paradigm Analyses of the four independent components (positive voxels only). Only significant results are reported.

Paradigm Class	*z-score*	*Paradigm Class*	*z-score*
**IC1**		**IC2**	
Reward	36.64	Semantic Monitor/Discrimination	35.48
Pain Monitor/Discrimination	19.78	Phonological Discrimination	19.28
Finger Tapping/Button Press	16.38	Reading (Covert)	18.65
Go/No-Go	16.23	Finger Tapping/Button Press	17.69
Face Monitor/Discrimination	13.72	Word Generation (Covert)	17.52
Semantic Monitor/Discrimination	11.29	Face Monitor/Discrimination	17.08
Delayed Match to Sample	11.24	n-back	16.54
Taste	10.99	Delayed Match to Sample	16.07
Visuospatial Attention	10.93	Word Generation (Overt)	14.13
n-back	10.35	Cued Explicit Recognition/Recall	14.11
Cued Explicit Recognition/Recall	9.97	Counting/Calculation	13.06
Task Switching	9.55	Encoding	12.55
Emotion Induction	9.25	Reward	12.40
Paired Associate Recall	9.13	Reading (Overt)	12.17
Stroop-Color	8.76	Paired Associate Recall	12.12
Counting/Calculation	8.75	Go/No-Go	11.71
Olfactory Monitor/Discrimination	8.58	Passive Listening	11.53
Film Viewing	8.35	Pitch Monitor/Discrimination	11.48
Word Generation (Covert)	8.24	Orthographic Discrimination	11.43
Tone Monitor/Discrimination	8.17	Film Viewing	11.40
Affective Pictures	8.06	Visuospatial Attention	11.37
Recitation/Repetition (Overt)	7.99	Reasoning/Problem Solving	10.62
Encoding	7.64	Task Switching	10.23
Phonological Discrimination	7.42	Naming (Covert)	10.16
Word Generation (Overt)	7.40	Music Comprehension	10.15
Classical Conditioning	7.32	Stroop-Color	10.04
Acupuncture	6.88	Theory of Mind	10.02
Wisconsin Card Sorting Test	6.67	Imagined Objects/Scenes	9.45
Music Comprehension	6.63	Emotion Induction	9.42
Passive Viewing	6.54	Tone Monitor/Discrimination	9.31
Reasoning/Problem Solving	6.39	Passive Viewing	8.35
Episodic Recall	6.16	Visual Object Identification	7.74
Orthographic Discrimination	5.84	Figurative Language	7.48
Reading (Overt)	5.65	Affective Pictures	7.13
Delay Discounting	5.57	Wisconsin Card Sorting Test	7.11

Flexion/Extension	5.21	Naming (Overt)	6.87
Tactile Monitor/Discrimination	5.14	Mental Rotation	6.82
Motor Learning	5.14	Recitation/Repetition (Overt)	6.51
Micturition	5.13	Pain Monitor/Discrimination	6.40
Chewing/Swallowing	5.11	Word Stem Completion (Overt)	5.80
Flanker	5.07	Recitation/Repetition (Covert)	5.74
Visual Object Identification	5.04	Music Production	5.64
Deception	4.88	Imagined Movement	5.42
Pitch Monitor/Discrimination	4.68	Lexical Decision	5.28
Saccades	4.68	Oddball Discrimination	5.26
Imagined Objects/Scenes	4.57	Delay Discounting	5.21
Transcranial Magnetic Stimulation	4.42	Flanker	5.13
Anti-Saccades	4.28	Taste	5.09
Reading (Covert)	4.26	Emotional Body Language Perception	5.01
Music Production	4.12	Episodic Recall	4.96
Tower of London	4.11	Deception	4.88
Estimation	3.92	Stroop-Other	4.78
Oddball Discrimination	3.83	Word Stem Completion (Covert)	4.60
Recitation/Repetition (Covert)	3.71	Word Imageability	4.26
Imagined Movement	3.69	Syntactic Discrimination	4.10
Sexual Arousal/Gratification	3.49	Saccades	3.80
Stroop-Other	3.48	Tactile Monitor/Discrimination	3.79
Lexical Decision	3.40	Sequence Recall/Learning	3.76
Gambling	3.40	Classical Conditioning	3.61
Stroop-Emotional	3.31	Divided Auditory Attention	3.46
**IC3**		**IC4**	
Emotion Induction	18.92	Theory of Mind	16.46
Face Monitor/Discrimination	17.40	Emotion Induction	8.23
Passive Viewing	14.17	Episodic Recall	8.16
Reward	14.16	Semantic Monitor/Discrimination	7.96
Semantic Monitor/Discrimination	13.47	Face Monitor/Discrimination	7.87
Film Viewing	12.56	Reward	5.09
Affective Pictures	11.95	Imagined Objects/Scenes	4.77
Cued Explicit Recognition/Recall	9.98	Competition/Cooperation	4.68
Theory of Mind	9.47	Reasoning/Problem Solving	4.59
Finger Tapping/Button Press	9.34	Film Viewing	4.28
Visuospatial Attention	9.03	Self-Reflection	4.18
Delayed Match to Sample	9.00	Cued Explicit Recognition/Recall	3.95

Go/No-Go	8.89
Encoding	8.88
Episodic Recall	8.75
Reading (Covert)	8.18
Word Generation (Covert)	8.09
Paired Associate Recall	7.63
Word Generation (Overt)	7.12
Emotional Body Language Perception	6.56
Pain Monitor/Discrimination	6.49
Task Switching	6.48
Imagined Objects/Scenes	6.41
Olfactory Monitor/Discrimination	6.37
Visual Object Identification	6.31
Taste	6.24
Counting/Calculation	5.80
n-back	5.65
Reasoning/Problem Solving	5.53
Classical Conditioning	5.10
Acupuncture	5.06
Reading (Overt)	4.97
Phonological Discrimination	4.96
Music Comprehension	4.82
Affective Words	4.53
Tone Monitor/Discrimination	4.17
Orthographic Discrimination	4.12
Naming (Covert)	4.08
Stroop-Color	3.69
Passive Listening	3.60
Pitch Monitor/Discrimination	3.58
Figurative Language	3.55
Lexical Decision	3.43
Wisconsin Card Sorting Test	3.37
Naming (Overt)	3.35
Stroop-Other	3.20

Passive Viewing	3.94
Acupuncture	3.89
Deception	3.87
Passive Listening	3.60
Delay Discounting	3.56
Paired Associate Recall	3.45
Word Generation (Covert)	3.42

Figure S11: Intersections of the significant paradigms found with the Paradigm Analysis of the four independent components.

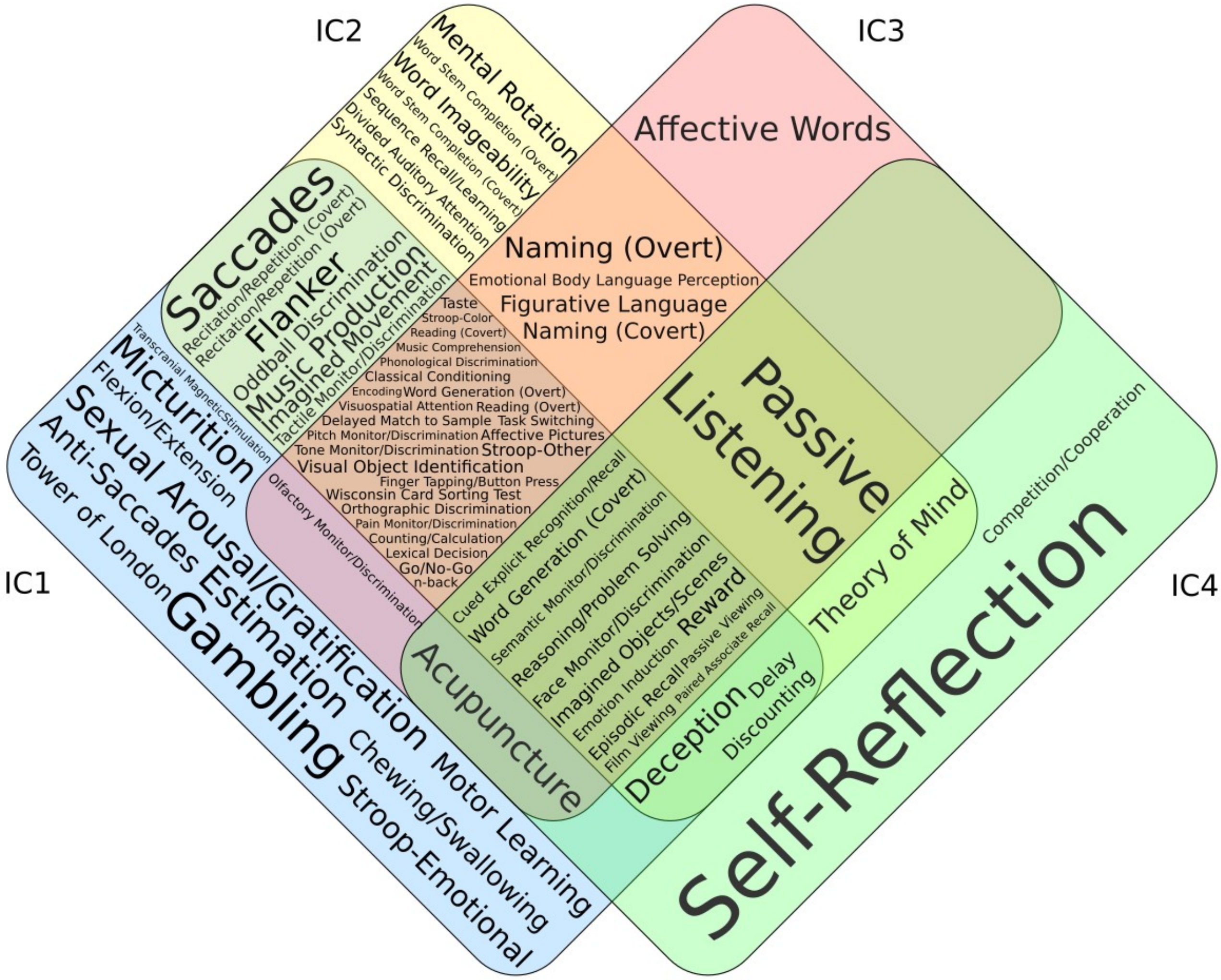

Figure S12: Independent Component Analyses with five components. Left: surface mapping of the voxel-wise scores. Right: bar graphs of the component loadings for each map.

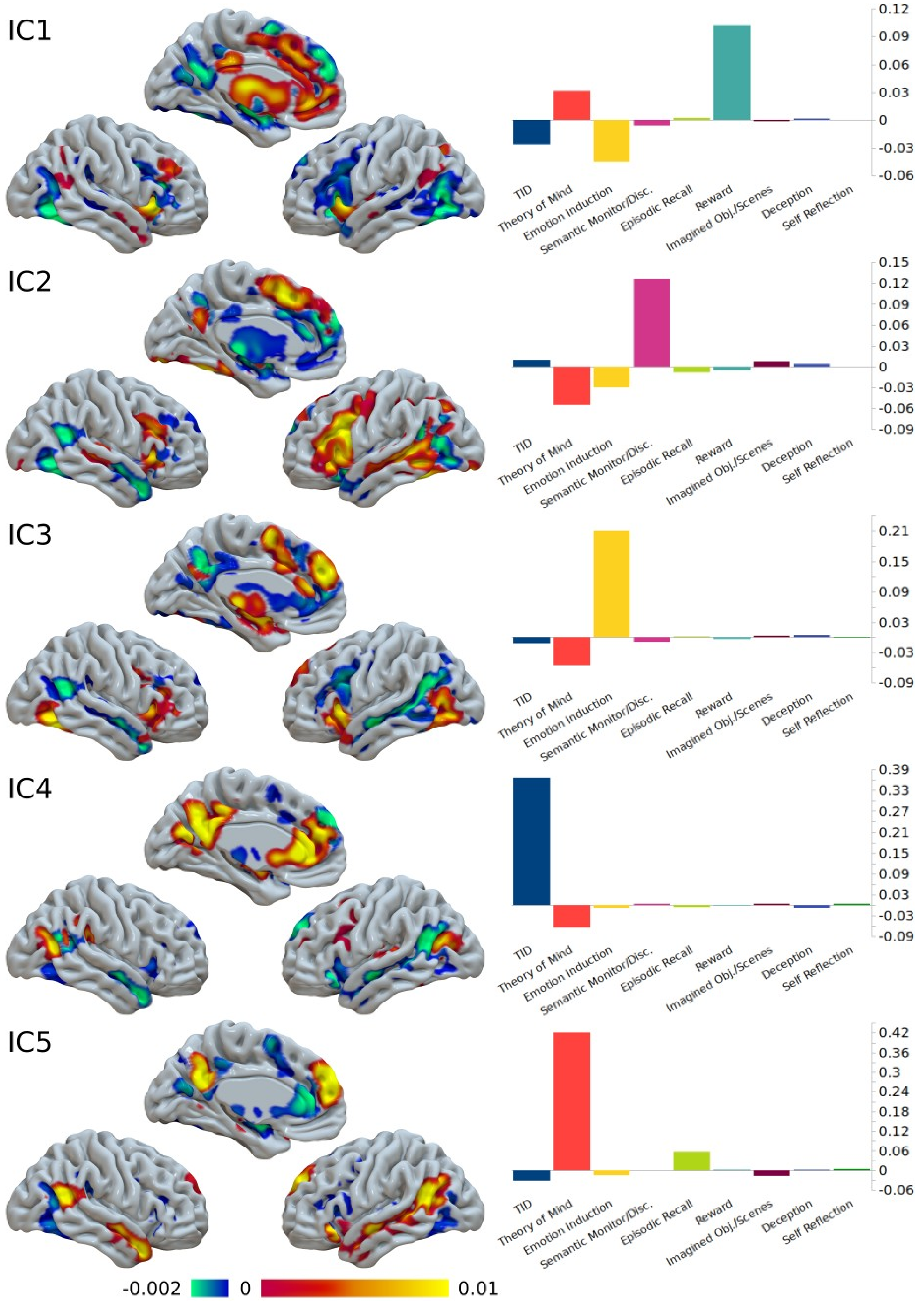

Figure S13: Independent Component Analyses with two (IC2.1 and IC2,2) and three (IC3.1, IC3.2 and IC3.3) components.

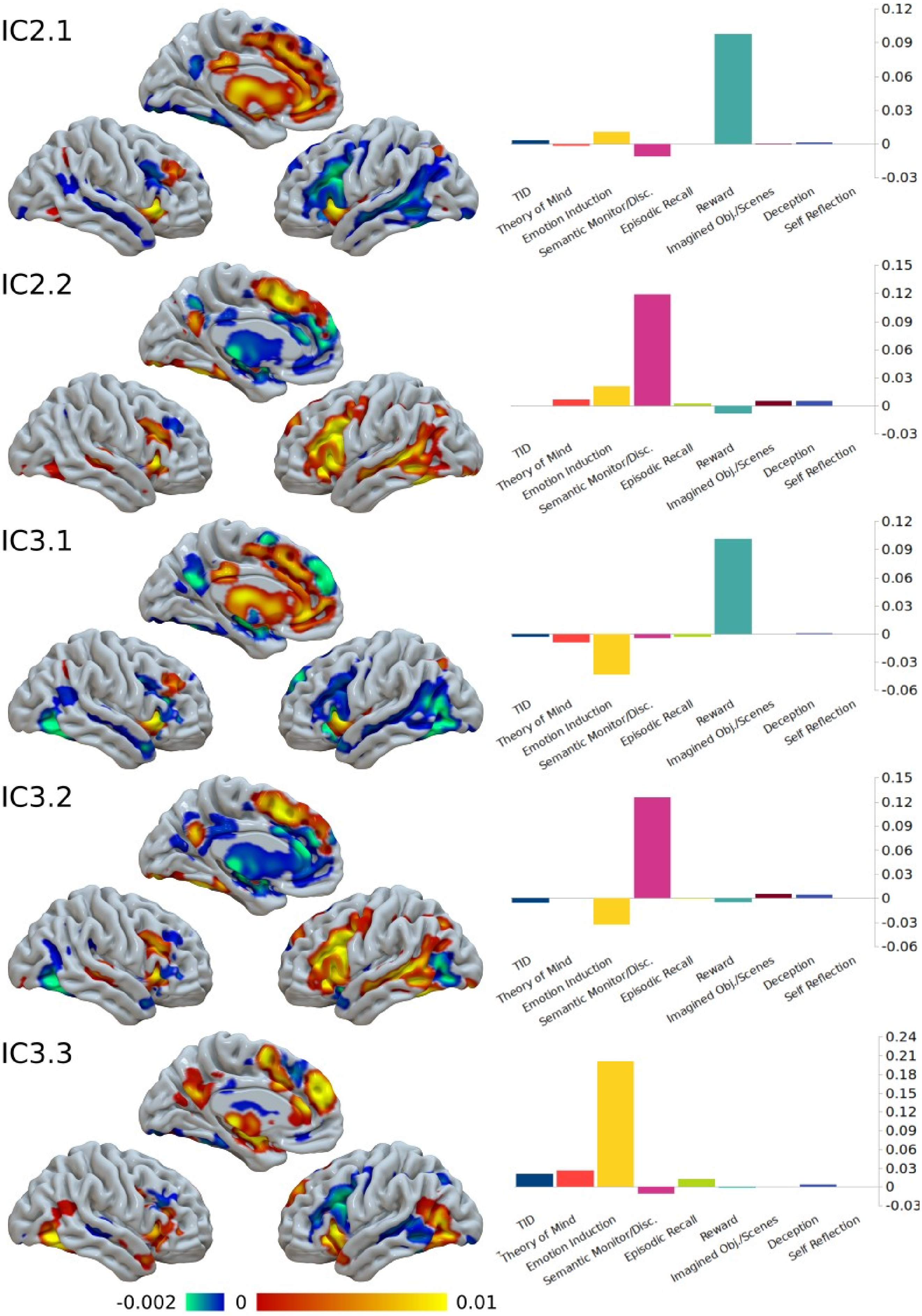

Testing the heteromodality of the principal gradients

To evaluate if the third principal gradient could be considered a map of heteromodal areas, we replicated the meta-analysis made by Margulies et al. (2016), using the Behavioral Analysis toolbox (Lancaster et al., 2012).

The given PG, converted into Talairach space, was divided in 20 ROIs including 5% of the map values distribution (e.g., from 0 to the 5th percentile, from the 5th to the 10th percentile, etc.), and each ROI was fed to the Behavioral Analysis plugin for Mango. The Behavioral Analysis is akin to the Paradigm Analysis (see Methods), but works on behavioral domains instead of experimental paradigms. The cognitive profile of the ROI is represented by the z-scores obtained by such analysis for the 60 behavioral categories, and it is illustrated in Fig. S14.

Figure S14: Behavioral Analyses z-scores of the ROIs containing the map distribution quintiles (QU) for principal gradient 1 and 3 by Margulies et al. (2016).

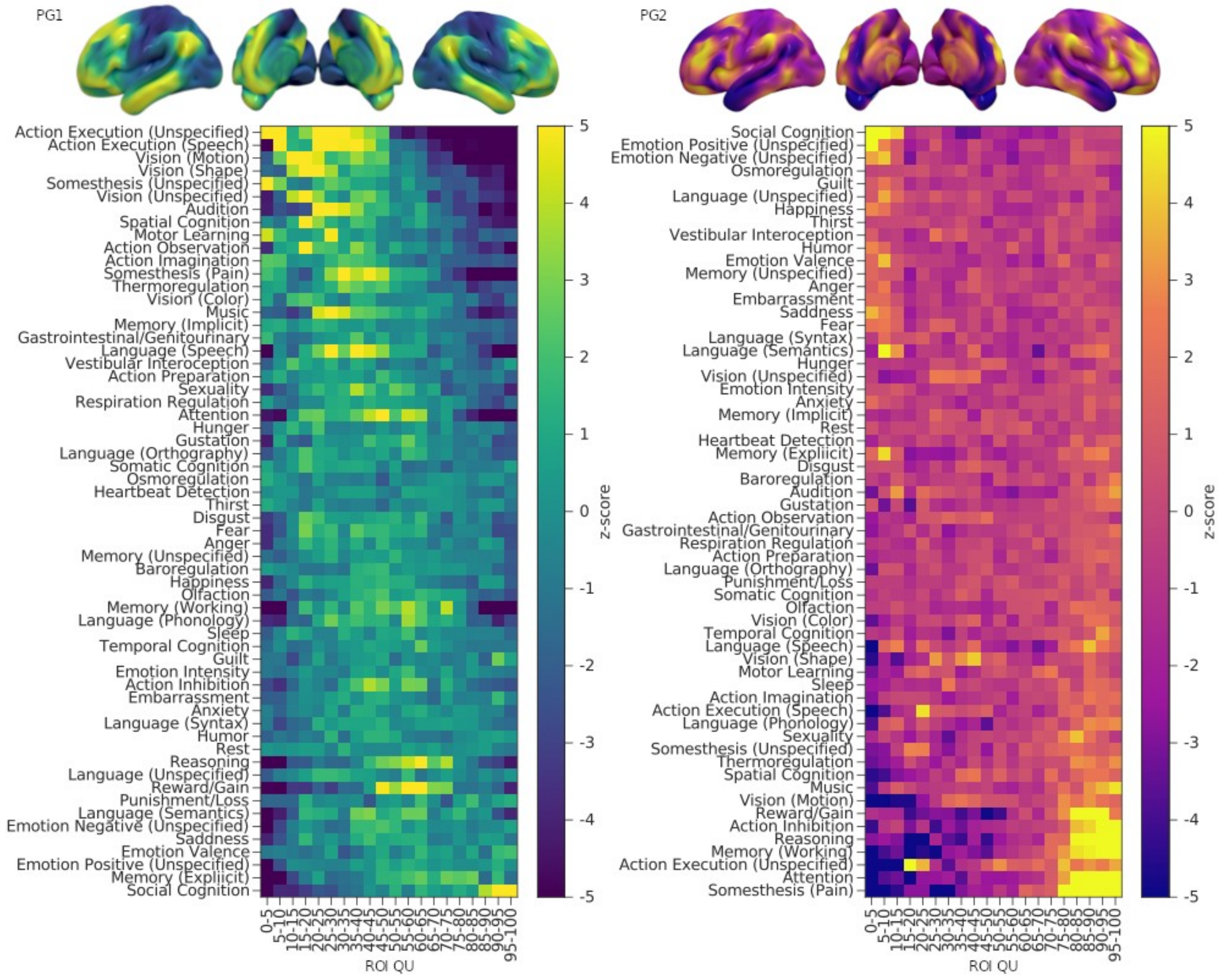

Table S4: Pearson's correlations between the nine ALE maps and four principal components (PC), and the two principal gradients (PG) by Margulies et al., and percentages of ALE and PC voxels > 0 overlapping with the PG voxels > 0.

	Pearson's *r*		Overlaps (positive voxels only)		
	**PG1**	**PG3**	**PG1**	**PG3**	**PG1 ∪ PG3**
Decrease	0.09	0.04	80%	56%	90%
Emotion Induction	0.05	-0.05	50%	36%	70%
Episodic Recall	0.11	-0.11	83%	12%	90%
Imagined Obj.-Scenes	0.04	0.03	65%	55%	86%
Reward	0.06	0.17	55%	65%	83%
Self Reflection	0.00	-0.02	39%	35%	73%
Semantic Monitor-Disc.	0.09	-0.03	52%	35%	66%
TID	0.14	-0.10	69%	17%	74%
ToM	0.19	-0.20	81%	3%	83%
PC1	0.08	0.14	52%	44%	71%
PC2	0.06	-0.11	50%	32%	64%
PC3	0.03	-0.11	57%	25%	71%
PC4	0.18	-0.15	72%	12%	76%

References

Desmond JE, Gabrieli JD, Wagner AD, et al (1997) Lobular patterns of cerebellar activation in verbal working-memory and finger-tapping tasks as revealed by functional MRI. J Neurosci 17:9675–9685

Lancaster JL, Laird AR, Eickhoff SB, et al (2012) Automated regional behavioral analysis for human brain images. Front Neuroinform 6:1–12. https://doi.org/10.3389/fninf.2012.00023

Margulies DS, Ghosh SS, Goulas A, et al (2016) Situating the default-mode network along a principal gradient of macroscale cortical organization. Proc Natl Acad Sci 113:12574–12579. https://doi.org/10.1073/pnas.1608282113
